# Genetically proxied blood pressure, vascular brain injury, and Alzheimer's disease pathology

**DOI:** 10.1002/alz.70515

**Published:** 2025-07-19

**Authors:** Iyas Daghlas, Michelle R. Caunca, Lincoln M. P. Shade, Malik Nassan, Kristine Yaffe, David W. Fardo, Dipender Gill

**Affiliations:** ^1^ Department of Neurology University of California San Francisco San Francisco California USA; ^2^ Department of Biostatistics, College of Public Health University of Kentucky Lexington Kentucky USA; ^3^ Mesulam Center for Cognitive Neurology and Alzheimer's Disease Northwestern University Chicago Illinois USA; ^4^ Department of Psychiatry and Behavioral Sciences University of California San Francisco San Francisco California USA; ^5^ Department of Epidemiology and Biostatistics University of California San Francisco San Francisco California USA; ^6^ Department of Epidemiology and Biostatistics, School of Public Health Imperial College London London UK

**Keywords:** Alzheimer's dementia, atherosclerosis, blood pressure, Mendelian randomization, systolic blood pressure, vascular dementia

## Abstract

**INTRODUCTION:**

Lower blood pressure (BP) is linked to reduced dementia risk, though it is uncertain whether this benefit stems solely from mitigating vascular brain injury (VBI) or also extends to directly influencing Alzheimer's disease (AD) pathology. We leveraged Mendelian randomization (MR) to assess whether lifelong lower BP is causally associated with neuropathological correlates of VBI and AD.

**METHODS:**

We identified genetic proxies for systolic and diastolic BP (*n *= 1,028,980) and applied them in MR analyses of post mortem neuropathological measures of VBI and AD (*n = *6363–7786).

**RESULTS:**

Genetically proxied lower systolic BP associated with reduced risk of all VBI measures, including atherosclerosis, arteriolosclerosis, gross infarcts, and microinfarcts. There was no evidence for associations between systolic BP and AD pathology, including measures of amyloid and tau pathology. Diastolic BP analyses yielded similar results.

**DISCUSSION:**

These findings suggest that BP‐lowering protects against dementia by mitigating VBI rather than by directly affecting AD pathology.

**Highlights:**

- It is uncertain whether blood pressure impacts dementia risk solely through its effects on blood vessel health, or whether it also impacts Alzheimer's disease (AD) pathology.
- We performed a Mendelian randomization study using genetic predictors of blood pressure applied to a unique autopsy study of neuropathological correlates of both vascular brain injury and AD pathology.
- We found evidence that genetically lowered blood pressure solely impacts vascular brain injury, with no effects on AD pathology.

## 1 INTRODUCTION

Findings from observational studies^1^, ^2^, population genetics research^3^, and clinical trials^4^, ^5^ support blood pressure (BP) lowering as an effective strategy for dementia prevention. The most common dementia subtypes are Alzheimer's disease (AD) and vascular dementia, each characterized by distinct neuropathological features^6^. While the effect of hypertension on vascular brain injury (VBI) is well established^7^, its influence on AD pathology is uncertain^8^, ^9^. Clarifying this relationship would advance the basic understanding of AD pathogenesis and would be of therapeutic relevance.

The uncertainty in the association of hypertension with AD stems in part from the challenge of definitively determining dementia subtypes in living patients, and from the frequent coexistence of VBI and AD pathology in the same patient^10^. These challenges undermine epidemiological investigations that rely on clinically diagnosed AD or vascular dementia as outcomes without confirmatory neuropathology. Although radiographic and laboratory‐based biomarkers offer more objective and direct measures of neuropathological phenotypes in living patients, epidemiological studies of these biomarkers have produced inconsistent results on their association with hypertension^8^, ^11^. Moreover, causal inference from these observational associations is limited by biases of unmeasured confounding and reverse causality^12^. Alternative epidemiologic approaches are therefore needed to address this question.

**FIGURE 1 alz70515-fig-0001:**
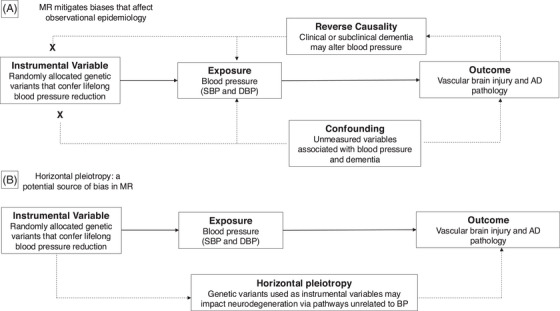
Directed acyclic graphs comparing causal pathways and biases in conventional observational epidemiological studies and in MR studies. A, Comparison of traditional observational studies versus MR approaches in assessing the relationship of blood pressure with neurodegeneration. MR leverages randomly inherited genetic variants as instrumental variables to mitigate biases related to confounding and reverse causation. B, The MR framework assumes that genetic proxies affect dementia pathology exclusively through blood pressure modification, rather than through independent biological pathways (horizontal pleiotropy). Sensitivity analyses evaluate the robustness of this assumption. AD, Alzheimer's disease; DBP, diastolic blood pressure; MR, Mendelian randomization; SBP, systolic blood pressure; VBI, vascular brain injury.

Naturally randomized data enhance the reliability of causal inference in observational studies. The Mendelian randomization (MR) paradigm uses genetic variants as proxies for health risk factors, leveraging their random allocation at gametogenesis^13^. This randomization minimizes confounding, while the fixed identity of germline variants ensures they are unaffected by disease development (Figure 1). MR has been used to investigate causal associations between health risk factors and dementia^14^, ^15^, though it has not been systematically applied to investigate neuropathological correlates of dementia. In this investigation, we applied the MR paradigm to post mortem neuropathological data to examine potential causal relationships between BP and VBI and AD pathology^16^.

## 2 METHODS

### 2.1 Ethics approval

This study used publicly available, deidentified data and was therefore exempt from institutional review board review. All contributing studies obtained informed consent from either the participant or a caregiver^16^, ^17^.

### 2.2 Study overview

The aim of this two‐sample MR study was to investigate potential causal relationships between genetically proxied lower BP and neuropathological correlates of VBI and AD. We use the term VBI to specifically denote neuropathological changes, distinguishing it from vascular dementia, which refers to a clinical phenotype. To conduct our analysis, we identified genetic proxies for systolic blood pressure (SBP) and diastolic blood pressure (DBP) and obtained their associations with neuropathological correlates of VBI and AD. MR analyses were then performed to estimate the effect of genetically proxied lower BP on each outcome.

### 2.3 Genetic associations with SBP and DBP

We obtained genetic associations with SBP and DBP from a 2024 genome‐wide association study (GWAS) meta‐analysis of 80 cohorts including 1,028,980 participants of European ancestry^17^. BP measurements were corrected for antihypertensive use by applying the established method of adding 15 mm Hg to SBP and 10 mm Hg to DBP^17^, ^18^. Genetic associations were adjusted for age at BP measurement, age^2^, sex, body mass index (BMI), and the top 10 principal components of ancestry. Beta coefficients for variants were reported as mm Hg change per effect allele.

**RESEARCH IN CONTEXT**

1. **Systematic review**: The authors reviewed the literature using PubMed. Hypertension has been linked to increased risk of vascular dementia and Alzheimer's disease (AD). However, there is no naturally randomized data in the literature that investigate the causal effects of blood pressure (BP) on the neuropathological changes that underlie these traits.
2. **Interpretation**: Findings from our Mendelian randomization analysis of post mortem neuropathological data suggest that BP lowering protects against dementia by mitigating vascular brain injury rather than by directly affecting AD pathology.
3. **Future directions**: Future investigations should assess non‐linear effects, characterize cutoffs, and investigate these associations in clinically relevant subgroups.

### 2.4 Genetic associations with neuropathological correlates of VBI and AD

We obtained genetic associations with autopsy‐based neuropathological measures of VBI and AD from a 2024 GWAS meta‐analysis of three data sources: National Alzheimer's Coordinating Center (NACC; *n *= 5940), Adult Changes in Thought (ACT; *n *= 681), and the Religious Orders Study and Rush Memory and Aging Project (ROSMAP; *n*  =  1183)^16^. The mean age at autopsy was 89 in ROSMAP (67% female)^19^, 89 in ACT (58% female)^20^, and 80 in NACC (50% female)^21^. Additional demographic details, distribution of clinical diagnoses, and methodologies for neuropathological analysis in these cohorts have been described previously^16^, ^19^, ^20^, ^21^. From the available neuropathological measures, we selected phenotypes[Fig alz70515-fig-0001] that could be definitively categorized as either correlates of VBI or AD (Table 1). These phenotypes were harmonized across the three datasets prior to genetic analysis, with most having directly comparable coding definitions. Ordinal phenotypes were analyzed using a proportional odds logistic mixed‐effects model, while binary phenotypes (presence of gross infarcts or microinfarcts) were analyzed using a logistic mixed‐effects model^16^. Genetic associations were adjusted for age at death, sex, cohort, and the top 10 genetic principal components of ancestry. These models report odds ratios (ORs) of the outcome per effect allele, indicating either increased severity for ordinal phenotypes or the relative likelihood of presence versus absence for binary phenotypes.

**TABLE 1:** Neuropathological correlates of vascular brain injury and AD examined in this study.

Phenotype	Category	Variable coding (category *n*)	Total *n*
Arteriolosclerosis	Vascular brain injury	Ordinal: none (1645) versus mild (2122) versus moderate (2067) versus severe (834)	6668
Atherosclerosis in circle of Willis	Vascular brain injury	Ordinal: none (1505) versus mild (2786) versus moderate (2231) versus severe (818)	7340
Gross infarcts	Vascular brain injury	Binary: absent (1766) versus present (5632)	7398
Microinfarcts	Vascular brain injury	Binary: absent (1839) versus present (5641)	7480
Braak NFT stage	AD—quantification of severity of neurofibrillary tangle (tau) pathology	Ordinal: absent (score 0)—to diffuse with large neuronal loss (score 6): 0 (110), 1 (342), 2 (585), 3 (896), 4 (1360), 5 (1947), 6 (2536)	7776
CERAD score	AD—quantification of neuritic plaque (Aβ) burden	Ordinal: none (1012) versus mild (786) versus moderate (1639) versus severe (4349)	7786
Diffuse plaques	AD pathology	Ordinal: none (693) versus mild (900) versus moderate (1233) versus severe (3537)	6363

Abbreviations: Aβ, amyloid beta; AD, Alzheimer's disease; CERAD, Consortium to Establish a Registry for Alzheimer's Disease; NFT, neurofibrillary tangle.

### 2.5 Selection of genetic proxies and data harmonization

The MR paradigm requires genetic variants to be strongly associated with the proxied exposures. Therefore, for each BP trait, we identified variants present in all GWAS datasets that met the genome‐wide statistical significance threshold (*P* < 5×10^−8^). To avoid bias due to pleiotropy (see below), we excluded variants in the apolipoprotein E (*APOE*) gene region (Chr19: 45,116,911–46,318,605)^15^. To identify independently inherited variants, we performed linkage disequilibrium (LD) clumping (*r* ^2 ^< 0.001 using a 1Mb window) using the 1000 Genomes European ancestry subset as our LD reference panel^22^. We extracted associations of these variants with the neuropathological traits and aligned exposure and outcome beta coefficients to the same effect allele. Palindromic variants were excluded when strand orientation could not be unambiguously determined (if minor allele frequency > 0.42)^22^. We report the mean *F* statistics as a measure of genetic proxy strength, considering 10 to represent the threshold for weak instruments^23^. We also report the percentage of variance explained of the respective BP exposures, which relates to the statistical power of the analysis.

### 2.6 MR analyses

We used the random effects inverse variance weighted method to estimate the effect of lifelong genetically lowered BP on each neuropathological outcome^22^. Effect estimates were scaled to a 10 mm Hg reduction in SBP or to a 5 mm Hg reduction in DBP, each approximately corresponding to a half‐standard deviation reduction in BP^24^; this permits comparison of SBP and DBP analyses on the same scale. To account for multiple comparisons, we applied a Bonferroni‐corrected statistical significance threshold of *P *< 0.007, conservatively adjusting for the seven outcomes. We did not apply additional correction for analyzing both BP traits given their strong pairwise correlation. Analyses were conducted using R version 4.4.2.

### 2.7 Sensitivity analyses

Genetic confounding in MR is minimized by matching ancestry groups between exposure and outcome datasets and by adjusting GWASs for genetic ancestry. However, MR analyses may be biased by pleiotropy, whereby genetic proxies influence the outcome through pathways independent of the exposure (Figure 1). Therefore, for significant associations in the primary analysis, we performed sensitivity analyses to assess the robustness of associations to violations of this MR assumption. First, we implemented the Cochran Q test for heterogeneity to evaluate whether the associations between proxy variants and the outcome exhibited greater variation than would be expected by chance alone. Second, we implemented the MR Egger intercept test for directional pleiotropy^25^. Third, we calculated MR estimates using the weighted median and MR Egger methods, which provide consistent causal estimates while relaxing assumptions about pleiotropy^25^, ^26^. Finally, to minimize bias due to potential reverse causality, we excluded genetic proxies within 500 kb of lead variants identified in the neuropathology GWAS^16^.

MR estimates may also be impacted by collider bias when genetic associations are adjusted for heritable covariates such as BMI^27^. To address this possibility, we repeated analyses using a GWAS of BP conducted in the UK Biobank cohort without BMI adjustment (*n *= 430,025)^28^. BP in this cohort was measured with an automated cuff using a standardized protocol. These genetic associations were adjusted for age, age^2^, sex, age x sex, age^2^ x sex, and the top 20 principal components of ancestry. Effect estimates were inverse normal transformed and therefore represent effects on the standard deviation scale; to match our primary analysis, we scaled MR estimates to reflect the effect of a half‐standard deviation change in BP (corresponding to a 10 mm Hg reduction in SBP or to a 5 mm Hg reduction in DBP).

## 3 RESULTS

We identified 831 independent variants to serve as proxies for SBP (Table S1 in supporting information). The mean *F* statistic was 70 (range 30–701), and the variants collectively explained 4.4% of the variance of SBP. We identified 826 independent variants to serve as proxies for DBP. The mean *F* statistic was 70 (range 30–829), and the variants collectively explained 6.7% of the variance of DBP (Table S2 in supporting information).

**FIGURE 2:**
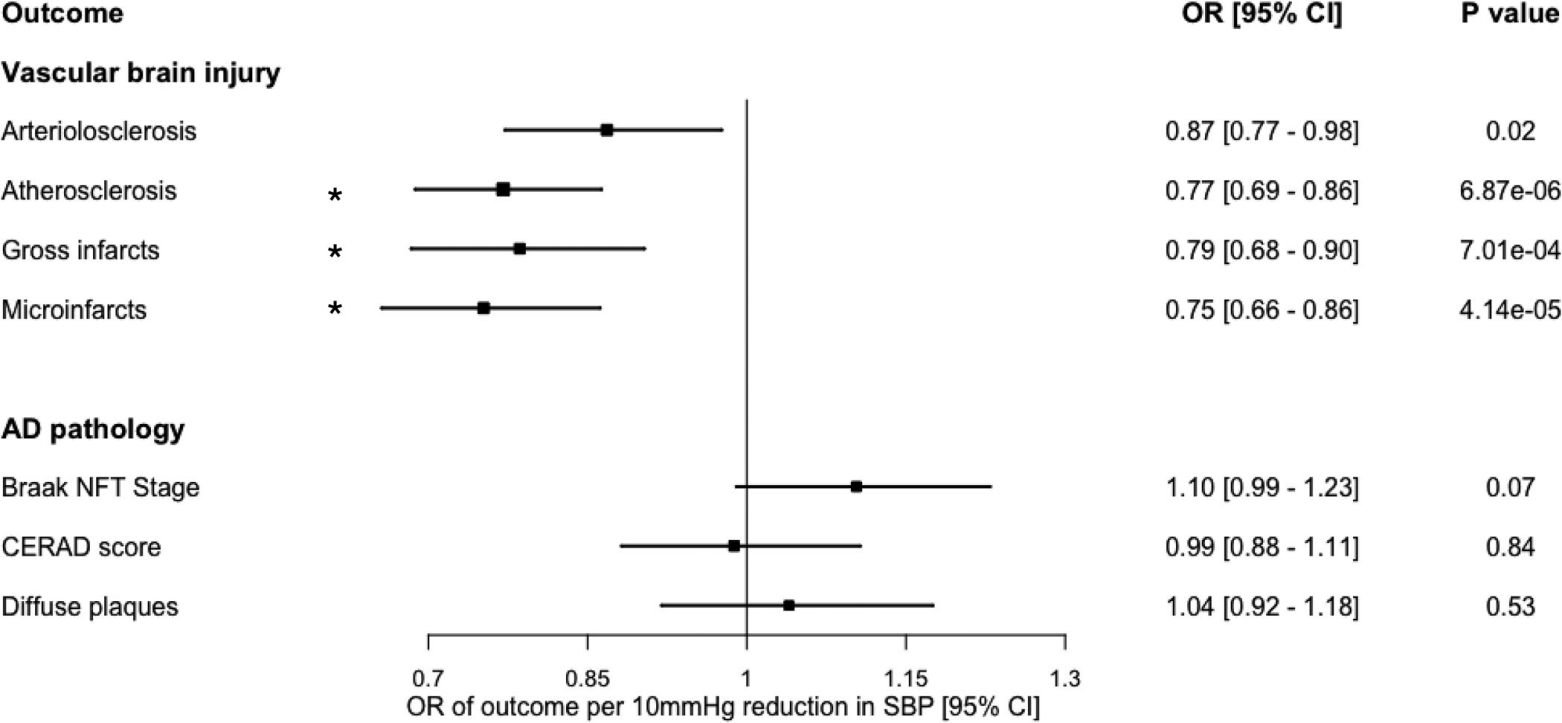
MR estimates for the association of genetically proxied lifelong lower SBP with neuropathological measures of vascular brain injury and AD pathology. Displayed are point estimates (boxes) and 95% CIs (horizontal lines). Estimates correspond to the effect of a SP reduction (proxied using 831 variants) of 10 mm Hg. Asterisks indicate estimates that surpassed the Bonferroni‐adjusted statistical significance threshold. AD, Alzheimer's disease; CERAD, Consortium to Establish a Registry for Alzheimer's Disease; CI, confidence interval; MR, Mendelian randomization; NFT, neurofibrillary tangle; OR, odds ratio; SBP, systolic blood pressure

Genetically proxied lower SBP was significantly associated with reduced severity of atherosclerosis in the circle of Willis (OR of category increase in atherosclerosis severity per 10 mm Hg lower SBP: 0.77, 95% confidence interval [CI] 0.69–0.86, *P = *6.87 × 10^−6^), reduced risk of gross infarcts (OR 0.79, 95% CI 0.68–0.90, *p *= 7.01 × 10^−4^), and reduced risk of microinfarcts (OR 0.75, 95% CI 0.66–0.86, *p *= 4.14 × 10^−5^; Figure 2). There was a nominal association with reduced severity of arteriolosclerosis (OR 0.87, 95% CI 0.77–0.98, *p *= 0.02). There were no significant associations between genetically proxied SBP and any neuropathological correlates of AD (all *P *> 0.05; Figure 2).

**FIGURE 3:**
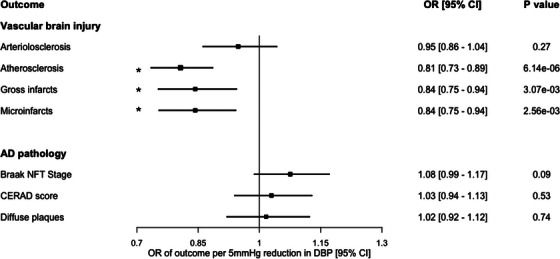
MR estimates for the association of genetically proxied lifelong lower DBP with neuropathological measures of vascular brain injury and AD pathology. Displayed are point estimates (boxes) and 95% CIs (horizontal lines). Estimates correspond to the effect of a DBP reduction (proxied using 826 variants) of 5 mm Hg. Asterisks indicate estimates that surpassed the Bonferroni‐adjusted statistical significance threshold. AD, Alzheimer's disease; CERAD, Consortium to Establish a Registry for Alzheimer's Disease; CI, confidence interval; DBP, diastolic blood pressure; MR, Mendelian randomization; NFT, neurofibrillary tangle; OR, odds ratio

Genetically proxied lower DBP was associated with protection from atherosclerosis (OR for category increase in atherosclerosis severity per 5 mm Hg lower DBP: 0.81, 95% CI 0.73–0.89, *p *= 6.14 × 10^−6^), gross infarcts (OR 0.84, 95% CI 0.75–0.94, *p *= 3.07 × 10^−3^), and microinfarcts (OR 0.84, 95% CI 0.75–0.94, *p *= 2.56 × 10^−3^; Figure 3). There was no significant association between genetically proxied DBP and arteriolosclerosis or any of the AD pathology measures (all *P *> 0.05; Figure 3).

Sensitivity analyses supported the primary analysis findings, with no significant evidence of bias due to pleiotropy based on Cochran Q and MR Egger intercept tests (Table S3 in supporting information). To mitigate potential bias due to reverse causality, we performed additional analyses excluding proxy variants located near rs2000660 in *COL4A1* (previously associated with atherosclerosis) and rs78909048 in *LZTS1* (previously associated with arteriolosclerosis). The exclusion of these variants did not materially alter the MR estimates, confirming the robustness of our findings (Table S3).

## 4 DISCUSSION

In this two‐sample MR study, we found that genetically proxied lifelong lower BP reduced the risk of multiple neuropathological correlates of VBI. By contrast, we found no evidence that lower BP was associated with any neuropathological correlates of AD. Taken together, this naturally randomized genetic evidence supports the notion that hypertension impacts brain health exclusively through effects on VBI, rather than through additional effects on AD pathology. Putative mechanisms for this vascular effect include blood vessel wall remodeling, microvascular rarefaction, endothelial cell injury and inflammation, and impaired neurovascular coupling^9^. The absence of BP effects on AD pathology underscores the need for ongoing development of targeted, mechanistic interventions for AD. These efforts should parallel optimization of individual and population‐level vascular health, as VBI and AD pathology additively increase all‐cause dementia risk^29^, ^30^.

Our study has several key strengths. The use of naturally randomized human genetic data within the MR framework mitigates bias due to residual confounding and reverse causality. We maximized statistical power by using strong genetic proxies from the largest available BP GWAS, yielding relatively precise effect estimates with narrow confidence intervals. Finally, the use of post mortem data enabled direct measurement of neuropathology, thus avoiding biases that impact analyses of clinically diagnosed dementia. Several limitations warrant consideration. First, these analyses were conducted using genetic data from individuals of European ancestry, potentially limiting generalizability to populations of other ancestral backgrounds. These MR analyses should be revisited in future studies as genetic associations with neuropathological phenotypes are identified in more diverse populations. Second, summary‐level MR analyses assume a linear relationship between exposure and outcome, and we therefore cannot exclude non‐linear effects of BP extremes. Although non‐linear MR methods have been developed, they require individual‐level data and are prone to false‐negative findings when applied to datasets with limited sample sizes. Third, survival or selection bias may have influenced our findings, as individuals who underwent neuropathological examination may not be representative of the general population. Fourth, variability in the subjective assessment of neuropathological phenotypes across contributing cohorts may have led to measurement error and attenuation of findings toward the null. Fifth, our MR estimates reflect effects of lifelong genetic influences on BP. Consequently, we cannot assess how these effects vary across different life stages, and these findings may not readily generalize to the effects of shorter term antihypertensive therapy. Sixth, given the use of summary‐level genetic data, we were unable to stratify by important subgroups such as age, sex, and *APOE* ε4 status. This limitation warrants further investigation in future analyses.

In conclusion, these human genetic data suggest that protective effects of BP lowering on dementia are primarily driven by mitigation of VBI, rather than through direct effects on AD pathology. This reinforces the importance of multifaceted therapeutic strategies in dementia prevention.

## CONFLICT OF INTEREST STATEMENT

DG is the chief executive officer of Sequoia Genetics, a private limited company that works with investors, pharma, biotech, and academia by performing research that leverages genetic data to help inform drug discovery and development. DG has interests in several biotechnology companies. All other authors (ID, MRC, LMPS, MN, KY, DWF) have no conflicts to declare. Author disclosures are available in the supporting information.

## CONSENT STATEMENT

All participants in the contributing genetic studies provided informed consent.

## Supporting information



Supporting information

Supporting information
