# Supplementary material for: Genetically proxied blood pressure, vascular brain injury, and Alzheimer's disease pathology
Source: Alzheimers Dement. 2025 Jul 19;21(7):e70515. doi: 10.1002/alz.70515 (PMC12276072; doi:10.1002/alz.70515)
Supplement: Supplementary file 1 — Supporting information [file ALZ-21-e70515-s001.docx]

**Supplementary Table 1. Genetic variants used to proxy effects of systolic blood pressure.**

Table is provided at end of document. EA: effect allele; EAF: effect allele frequency; OA: other allele; SE: standard error; SNP: single nucleotide polymorphism.

**Supplementary Table 2. Genetic variants used to proxy effects of diastolic blood pressure.**

Table is provided at end of document. EA: effect allele; EAF: effect allele frequency; OA: other allele; SE: standard error; SNP: single nucleotide polymorphism.

**Supplementary Table 3. Findings from MR sensitivity analyses for association of genetically proxied systolic blood pressure (SBP) with neuropathological correlates of vascular brain injury.** We performed sensitivity analyses for significant associations in our primary analysis. MR estimates reported from the UKB correspond to a half-standard deviation reduction in SBP (corresponding approximately to 10mmHg) and DBP (corresponding approximately to 5mmHg). Estimates in all other analyses correspond to a 10mmHg reduction in SBP and 5mmHg reduction in DBP. After excluding variants near *COL4A1* and *LZTS1* genes, 830 variants remained for systolic blood pressure analyses and 823 variants for diastolic blood pressure analyses. CI: confidence interval; IVW: inverse-variance weighted; UKB: UK Biobank.

| **Phenotype** | | **Odds ratio [95% CI]** | | | | **Global tests for pleiotropy** | |
| --- | --- | --- | --- | --- | --- | --- | --- |
| **Exposure** | **Outcome** | **UKB BP GWAS**  **(IVW Method)** | **Weighted Median** | **MR Egger** | **Excluding *COL4A1* and *LZTS1*** | **Cochrane’s Q for heterogeneity** | **Egger intercept test** |
| SBP | Gross infarcts | 0.77  [0.66-0.90] | 0.75  [0.60-0.94] | 0.64  [0.44-0.92] | 0.79  [0.68-0.90] | 0.08 | 0.23 |
|  | Microinfarcts | 0.79  [0.68-0.91] | 0.76  [0.61-0.94] | 0.60  [0.42-0.85] | 0.75  [0.66-0.86] | 0.18 | 0.17 |
|  | Arteriolosclerosis | 0.88  [0.77-1.01] | 0.92  [0.76-1.10] | 0.99  [0.73-1.35] | 0.87  [0.77-0.98] | 0.36 | 0.35 |
|  | Atherosclerosis | 0.80  [0.71-0.91] | 0.77  [0.65-0.91] | 0.73  [0.55-0.99] | 0.77  [0.69-0.86] | 0.25 | 0.73 |
| DBP | Gross infarcts | 0.87  [0.75-1.01] | 0.68  [0.48-0.97] | 0.61  [0.34-1.09] | 0.85  [0.76-0.95] | 0.06 | 0.58 |
|  | Microinfarcts | 0.89  [0.77-1.03] | 0.64  [0.44-0.92] | 0.59  [0.34-1.04] | 0.84  [0.75-0.94] | 0.11 | 0.50 |
|  | Atherosclerosis | 0.83  [0.73-0.94] | 0.61  [0.46-0.82] | 0.51  [0.32-0.82] | 0.81  [0.74-0.89] | 0.12 | 0.27 |

**Supplementary Table 1**. **Genetic variants used to proxy effects of systolic blood pressure.**

Table is provided at end of document. EA: effect allele; EAF: effect allele frequency; OA: other allele; SE: standard error; SNP: single nucleotide polymorphism.

| **SNP** | **EA** | **OA** | **EAF** | **Beta** | **SE** | **P** |
| --- | --- | --- | --- | --- | --- | --- |
| rs10048404 | T | C | 0.3661 | -0.2161 | 0.0266 | 4.63E-16 |
| rs10052777 | C | T | 0.3961 | 0.2841 | 0.0248 | 2.73E-30 |
| rs10066799 | T | G | 0.2808 | 0.1841 | 0.0269 | 7.96E-12 |
| rs10069690 | T | C | 0.2585 | 0.2762 | 0.0286 | 4.43E-22 |
| rs1009017 | C | T | 0.744 | 0.2061 | 0.0275 | 6.64E-14 |
| rs1010064 | C | A | 0.1848 | -0.3232 | 0.031 | 1.97E-25 |
| rs10151519 | A | G | 0.1926 | -0.2005 | 0.0308 | 7.05E-11 |
| rs10172510 | A | G | 0.439 | 0.1342 | 0.0242 | 2.85E-08 |
| rs10184428 | A | C | 0.5325 | 0.4417 | 0.0244 | 5.67E-73 |
| rs10188388 | T | C | 0.4123 | -0.198 | 0.0248 | 1.42E-15 |
| rs10196039 | T | C | 0.5413 | -0.1602 | 0.0247 | 9.02E-11 |
| rs10204932 | A | G | 0.0582 | 0.3143 | 0.0524 | 2.01E-09 |
| rs10224210 | C | T | 0.2805 | 0.353 | 0.0269 | 3.16E-39 |
| rs10233127 | A | T | 0.1098 | 0.2945 | 0.0403 | 2.82E-13 |
| rs10409243 | T | C | 0.5866 | -0.2457 | 0.0253 | 2.34E-22 |
| rs1043069 | G | T | 0.382 | -0.1817 | 0.0252 | 5.56E-13 |
| rs10433642 | G | A | 0.1569 | -0.2306 | 0.0333 | 4.56E-12 |
| rs10437954 | A | G | 0.9035 | -0.348 | 0.0419 | 9.44E-17 |
| rs1044822 | T | C | 0.1472 | -0.2291 | 0.0343 | 2.29E-11 |
| rs10468291 | A | C | 0.5695 | -0.164 | 0.0245 | 2.31E-11 |
| rs10477399 | A | G | 0.1907 | -0.3311 | 0.0314 | 4.59E-26 |
| rs10493891 | T | C | 0.2805 | -0.2223 | 0.0269 | 1.50E-16 |
| rs1057040 | A | G | 0.528 | -0.2048 | 0.0239 | 1.23E-17 |
| rs10732433 | T | C | 0.4237 | 0.1411 | 0.0245 | 8.14E-09 |
| rs10759426 | C | T | 0.1275 | 0.3446 | 0.0362 | 1.69E-21 |
| rs10761530 | C | T | 0.5017 | -0.1643 | 0.0241 | 8.86E-12 |
| rs10766318 | T | C | 0.7951 | -0.4225 | 0.0298 | 1.32E-45 |
| rs10766533 | A | T | 0.7086 | 0.1556 | 0.0268 | 6.26E-09 |
| rs10776752 | T | G | 0.078 | 0.7251 | 0.046 | 7.28E-56 |
| rs10777213 | A | G | 0.5252 | -0.1695 | 0.024 | 1.59E-12 |
| rs10782230 | A | G | 0.4826 | 0.192 | 0.0243 | 2.56E-15 |
| rs10789465 | C | T | 0.4365 | 0.1354 | 0.0243 | 2.46E-08 |
| rs10793931 | C | G | 0.3585 | 0.1414 | 0.0252 | 2.08E-08 |
| rs10817535 | G | T | 0.5933 | -0.198 | 0.0247 | 1.21E-15 |
| rs10820855 | C | T | 0.3149 | 0.1495 | 0.0265 | 1.69E-08 |
| rs10832778 | G | C | 0.6206 | -0.3181 | 0.0249 | 2.79E-37 |
| rs10833746 | A | G | 0.5129 | -0.1473 | 0.0245 | 1.73E-09 |
| rs10835161 | T | G | 0.594 | -0.1704 | 0.0252 | 1.26E-11 |
| rs10835313 | A | G | 0.505 | -0.2029 | 0.0245 | 1.31E-16 |
| rs10842709 | G | T | 0.3302 | -0.2082 | 0.0256 | 4.28E-16 |
| rs10845621 | T | C | 0.2807 | -0.2493 | 0.0274 | 8.76E-20 |
| rs10851885 | G | A | 0.2447 | 0.2119 | 0.029 | 2.54E-13 |
| rs10863593 | C | T | 0.3231 | -0.1891 | 0.0257 | 2.09E-13 |
| rs10869543 | A | T | 0.7595 | -0.1702 | 0.0291 | 5.15E-09 |
| rs10876531 | C | A | 0.294 | -0.3653 | 0.0268 | 3.59E-42 |
| rs10888388 | T | C | 0.5021 | 0.1349 | 0.0241 | 2.26E-08 |
| rs10904910 | A | C | 0.31 | 0.1551 | 0.026 | 2.56E-09 |
| rs10924380 | A | G | 0.1645 | -0.1837 | 0.0327 | 1.99E-08 |
| rs10941043 | G | T | 0.2879 | 0.192 | 0.0265 | 4.37E-13 |
| rs10952731 | G | A | 0.3058 | -0.1519 | 0.0261 | 5.89E-09 |
| rs10980141 | C | G | 0.1735 | -0.2021 | 0.0335 | 1.51E-09 |
| rs11000132 | T | C | 0.1644 | -0.1931 | 0.0328 | 3.78E-09 |
| rs11006779 | C | T | 0.5279 | 0.1821 | 0.0241 | 4.14E-14 |
| rs11026590 | C | A | 0.0746 | 0.3386 | 0.0473 | 8.18E-13 |
| rs11031051 | C | A | 0.3126 | 0.1833 | 0.0261 | 2.01E-12 |
| rs11037809 | G | A | 0.3035 | -0.1532 | 0.0263 | 5.51E-09 |
| rs11042089 | C | T | 0.249 | -0.1772 | 0.028 | 2.60E-10 |
| rs11042557 | C | T | 0.3784 | 0.1876 | 0.0251 | 7.34E-14 |
| rs1105123 | T | C | 0.7268 | 0.1495 | 0.0272 | 3.85E-08 |
| rs11064 | G | A | 0.2728 | -0.1547 | 0.0273 | 1.39E-08 |
| rs11070245 | G | T | 0.5311 | 0.1989 | 0.0242 | 1.88E-16 |
| rs11074093 | T | C | 0.5869 | -0.1978 | 0.0246 | 9.42E-16 |
| rs11076401 | A | G | 0.3054 | -0.1705 | 0.0262 | 7.50E-11 |
| rs11080134 | G | A | 0.3531 | 0.144 | 0.0255 | 1.55E-08 |
| rs11087740 | C | T | 0.4891 | 0.1326 | 0.0242 | 4.31E-08 |
| rs11105375 | A | T | 0.1679 | -0.7405 | 0.0325 | 2.94E-115 |
| rs11112548 | T | A | 0.0438 | -0.4494 | 0.0626 | 7.15E-13 |
| rs11117882 | G | A | 0.2356 | 0.2333 | 0.0285 | 2.44E-16 |
| rs11123059 | A | G | 0.5683 | 0.1342 | 0.0243 | 3.47E-08 |
| rs11125883 | C | A | 0.3722 | -0.1502 | 0.0249 | 1.68E-09 |
| rs11170390 | A | G | 0.0608 | -0.2977 | 0.0525 | 1.39E-08 |
| rs11178008 | T | C | 0.2381 | 0.1659 | 0.0281 | 3.48E-09 |
| rs111833760 | A | G | 0.0491 | 0.3357 | 0.056 | 2.05E-09 |
| rs11187838 | A | G | 0.4358 | -0.358 | 0.0242 | 1.44E-49 |
| rs11190709 | A | G | 0.8869 | 0.493 | 0.0383 | 5.59E-38 |
| rs11191531 | C | G | 0.0818 | -1.0058 | 0.0446 | 1.74E-112 |
| rs11210866 | C | T | 0.444 | 0.2105 | 0.0249 | 2.61E-17 |
| rs11214436 | T | G | 0.3908 | -0.1486 | 0.025 | 2.75E-09 |
| rs11222084 | T | A | 0.3607 | 0.3286 | 0.0255 | 5.08E-38 |
| rs11229555 | T | G | 0.2413 | -0.275 | 0.0285 | 5.50E-22 |
| rs11241305 | A | C | 0.4894 | 0.2515 | 0.0241 | 1.58E-25 |
| rs11247642 | C | T | 0.8219 | 0.2262 | 0.035 | 1.01E-10 |
| rs11249891 | T | C | 0.4736 | 0.2609 | 0.0243 | 7.46E-27 |
| rs11252324 | T | G | 0.0781 | -0.3242 | 0.0454 | 9.13E-13 |
| rs112684153 | C | T | 0.0735 | -0.3396 | 0.0498 | 9.24E-12 |
| rs112912733 | A | C | 0.0983 | -0.3106 | 0.0416 | 8.06E-14 |
| rs113086489 | T | C | 0.5508 | 0.3023 | 0.0249 | 7.60E-34 |
| rs113134141 | G | A | 0.102 | 0.2223 | 0.04 | 2.76E-08 |
| rs113252144 | T | C | 0.0715 | -0.2891 | 0.0529 | 4.57E-08 |
| rs1133400 | G | A | 0.2132 | 0.2622 | 0.0298 | 1.24E-18 |
| rs113393496 | A | G | 0.1955 | -0.172 | 0.0308 | 2.47E-08 |
| rs113402169 | T | C | 0.2102 | -0.1636 | 0.0298 | 4.20E-08 |
| rs1138293 | T | C | 0.1964 | -0.1834 | 0.0305 | 1.77E-09 |
| rs113892147 | A | G | 0.0454 | -0.3259 | 0.0589 | 3.14E-08 |
| rs113993899 | T | G | 0.2175 | 0.1872 | 0.0304 | 7.09E-10 |
| rs114183675 | T | C | 0.0425 | 0.456 | 0.0618 | 1.65E-13 |
| rs1148559 | T | C | 0.1934 | 0.23 | 0.0306 | 5.53E-14 |
| rs11571376 | G | C | 0.2951 | 0.1985 | 0.0267 | 1.10E-13 |
| rs11579440 | C | T | 0.151 | -0.2186 | 0.0342 | 1.68E-10 |
| rs11592107 | A | G | 0.3079 | 0.2384 | 0.0261 | 6.13E-20 |
| rs11606658 | C | T | 0.4553 | -0.1525 | 0.0241 | 2.53E-10 |
| rs11614730 | A | G | 0.6703 | -0.2157 | 0.0265 | 3.68E-16 |
| rs11615334 | T | C | 0.2581 | -0.2241 | 0.0283 | 2.28E-15 |
| rs11622562 | T | C | 0.3304 | -0.1572 | 0.0256 | 7.71E-10 |
| rs11636952 | C | T | 0.6807 | -0.4329 | 0.026 | 3.11E-62 |
| rs11638729 | A | G | 0.5554 | -0.1635 | 0.0249 | 5.14E-11 |
| rs11650262 | C | T | 0.0418 | -0.3769 | 0.0632 | 2.48E-09 |
| rs11684301 | G | A | 0.2125 | 0.1641 | 0.03 | 4.55E-08 |
| rs11690717 | T | G | 0.3661 | -0.1636 | 0.026 | 3.32E-10 |
| rs11696608 | T | C | 0.0357 | -0.3809 | 0.0665 | 1.02E-08 |
| rs117037316 | G | C | 0.0686 | 0.3414 | 0.0511 | 2.28E-11 |
| rs117472357 | T | C | 0.0413 | 0.4611 | 0.0634 | 3.60E-13 |
| rs11761199 | G | A | 0.4538 | -0.1749 | 0.0242 | 5.30E-13 |
| rs11771323 | A | G | 0.3011 | -0.1475 | 0.0262 | 1.76E-08 |
| rs117777118 | A | G | 0.0402 | -0.4587 | 0.0764 | 1.97E-09 |
| rs117913411 | A | T | 0.0375 | 0.5019 | 0.0675 | 1.06E-13 |
| rs118081085 | T | C | 0.0429 | 0.4415 | 0.0653 | 1.38E-11 |
| rs11856577 | C | T | 0.4672 | 0.2339 | 0.0245 | 1.12E-21 |
| rs11876341 | G | A | 0.3092 | 0.2181 | 0.0267 | 3.47E-16 |
| rs11896222 | G | C | 0.0923 | -0.2527 | 0.0424 | 2.57E-09 |
| rs11933087 | T | A | 0.357 | 0.1547 | 0.0266 | 5.71E-09 |
| rs11956654 | G | A | 0.2586 | -0.1782 | 0.0274 | 7.65E-11 |
| rs11977526 | A | G | 0.4047 | -0.3294 | 0.0246 | 9.67E-41 |
| rs11981330 | G | A | 0.3458 | -0.1558 | 0.0257 | 1.36E-09 |
| rs11988716 | G | A | 0.1344 | 0.211 | 0.0358 | 3.62E-09 |
| rs11998678 | T | C | 0.473 | 0.2504 | 0.0243 | 5.77E-25 |
| rs12035750 | C | T | 0.3713 | 0.2483 | 0.0252 | 6.88E-23 |
| rs12039740 | T | C | 0.1061 | -0.2291 | 0.0401 | 1.14E-08 |
| rs12042924 | C | T | 0.4665 | 0.1658 | 0.0245 | 1.19E-11 |
| rs12088448 | C | A | 0.3527 | 0.1767 | 0.0256 | 5.65E-12 |
| rs12102426 | G | T | 0.428 | -0.2764 | 0.0243 | 4.72E-30 |
| rs12114418 | G | A | 0.2359 | 0.2348 | 0.0288 | 3.49E-16 |
| rs12138150 | T | C | 0.3938 | -0.2076 | 0.0247 | 4.50E-17 |
| rs1214761 | G | A | 0.6655 | -0.3278 | 0.0257 | 2.18E-37 |
| rs12149258 | G | A | 0.1591 | -0.2351 | 0.0331 | 1.23E-12 |
| rs12153395 | A | G | 0.1097 | -0.288 | 0.0398 | 4.79E-13 |
| rs12185628 | C | T | 0.2185 | 0.1701 | 0.0295 | 7.92E-09 |
| rs12192157 | T | C | 0.5487 | 0.1772 | 0.0248 | 9.23E-13 |
| rs12192632 | G | C | 0.3893 | 0.169 | 0.0249 | 1.08E-11 |
| rs12192720 | A | G | 0.2874 | -0.1805 | 0.0268 | 1.70E-11 |
| rs12199746 | A | G | 0.595 | -0.1528 | 0.0247 | 6.16E-10 |
| rs1220128 | C | G | 0.8506 | 0.2115 | 0.034 | 4.81E-10 |
| rs12243365 | T | G | 0.2043 | -0.1785 | 0.0305 | 5.11E-09 |
| rs12258967 | G | C | 0.2972 | -0.5456 | 0.027 | 1.43E-90 |
| rs12291225 | A | C | 0.2567 | 0.1711 | 0.0284 | 1.60E-09 |
| rs1229984 | C | T | 0.9605 | 0.5988 | 0.0897 | 2.45E-11 |
| rs12343206 | G | A | 0.5884 | 0.1754 | 0.0253 | 3.79E-12 |
| rs12343516 | C | A | 0.5818 | -0.1858 | 0.0243 | 2.11E-14 |
| rs1234422 | A | T | 0.1873 | -0.1838 | 0.0309 | 2.77E-09 |
| rs12360330 | T | C | 0.1388 | 0.2398 | 0.0353 | 1.09E-11 |
| rs12378485 | T | G | 0.6519 | -0.185 | 0.0262 | 1.79E-12 |
| rs12419342 | T | C | 0.7029 | 0.1909 | 0.0266 | 7.23E-13 |
| rs12454712 | C | T | 0.3751 | -0.1999 | 0.026 | 1.35E-14 |
| rs1245811 | C | T | 0.6035 | -0.1584 | 0.0254 | 4.22E-10 |
| rs12459965 | T | C | 0.262 | -0.1949 | 0.0278 | 2.49E-12 |
| rs12463045 | C | T | 0.8291 | -0.2533 | 0.0334 | 3.62E-14 |
| rs12471513 | T | C | 0.3247 | 0.1626 | 0.0271 | 2.01E-09 |
| rs12494446 | A | G | 0.6288 | 0.2166 | 0.0249 | 3.15E-18 |
| rs1250259 | A | T | 0.7383 | -0.3031 | 0.0326 | 1.32E-20 |
| rs12509595 | C | T | 0.2915 | 0.7041 | 0.0266 | 5.97E-155 |
| rs12511987 | G | T | 0.178 | 0.207 | 0.0321 | 1.08E-10 |
| rs12514884 | A | G | 0.1579 | 0.1851 | 0.0334 | 2.90E-08 |
| rs12535680 | G | A | 0.3357 | 0.1807 | 0.0267 | 1.27E-11 |
| rs12543882 | T | C | 0.4587 | 0.1358 | 0.0241 | 1.66E-08 |
| rs12593120 | A | G | 0.1469 | 0.2139 | 0.0342 | 3.79E-10 |
| rs12619388 | A | C | 0.384 | 0.1383 | 0.0246 | 1.94E-08 |
| rs12623637 | G | A | 0.4452 | 0.1548 | 0.0245 | 2.48E-10 |
| rs12627514 | G | C | 0.2879 | 0.2717 | 0.0276 | 6.55E-23 |
| rs12638085 | A | T | 0.3496 | 0.2062 | 0.0261 | 2.80E-15 |
| rs12638862 | G | A | 0.263 | -0.2807 | 0.0275 | 1.73E-24 |
| rs12639622 | C | T | 0.1853 | -0.1918 | 0.0316 | 1.34E-09 |
| rs12661036 | C | T | 0.2267 | 0.1671 | 0.0304 | 3.96E-08 |
| rs12668436 | C | T | 0.2464 | 0.1732 | 0.028 | 6.42E-10 |
| rs12697889 | A | G | 0.4629 | -0.2154 | 0.0241 | 4.13E-19 |
| rs12716337 | C | T | 0.3806 | -0.4027 | 0.0251 | 5.79E-58 |
| rs12755444 | G | A | 0.285 | -0.2359 | 0.0269 | 1.70E-18 |
| rs1275980 | T | C | 0.6114 | -0.5164 | 0.0246 | 3.31E-98 |
| rs12807979 | C | A | 0.1009 | -0.238 | 0.0431 | 3.28E-08 |
| rs12895569 | C | T | 0.3605 | 0.1637 | 0.0251 | 6.76E-11 |
| rs12906962 | C | T | 0.326 | 0.2322 | 0.0261 | 5.48E-19 |
| rs1290933 | A | C | 0.6902 | -0.237 | 0.0261 | 1.20E-19 |
| rs12909648 | A | G | 0.4821 | 0.2115 | 0.0241 | 1.71E-18 |
| rs12920764 | T | C | 0.6068 | -0.1658 | 0.025 | 3.50E-11 |
| rs12922061 | T | C | 0.2284 | 0.1681 | 0.0293 | 1.01E-08 |
| rs12936625 | C | T | 0.6254 | 0.2191 | 0.025 | 1.67E-18 |
| rs12944684 | C | G | 0.3286 | 0.2812 | 0.0257 | 6.93E-28 |
| rs12955644 | G | C | 0.1792 | -0.3147 | 0.0324 | 2.59E-22 |
| rs12978472 | G | C | 0.1229 | -0.6848 | 0.0386 | 1.91E-70 |
| rs12980034 | C | T | 0.837 | -0.2005 | 0.0342 | 4.49E-09 |
| rs12981074 | G | A | 0.7344 | -0.1635 | 0.0273 | 2.08E-09 |
| rs13015703 | T | C | 0.2553 | 0.1718 | 0.0281 | 9.16E-10 |
| rs13022015 | C | A | 0.1847 | -0.173 | 0.0312 | 3.08E-08 |
| rs13043895 | C | T | 0.3303 | 0.1584 | 0.0254 | 4.65E-10 |
| rs13061156 | T | C | 0.0602 | -0.3129 | 0.0515 | 1.22E-09 |
| rs13106151 | A | G | 0.364 | -0.1479 | 0.025 | 3.10E-09 |
| rs13107325 | T | C | 0.0764 | -0.7456 | 0.0461 | 6.00E-59 |
| rs13118687 | A | G | 0.4636 | -0.2634 | 0.0244 | 4.13E-27 |
| rs13128814 | A | G | 0.5213 | -0.1842 | 0.0246 | 6.44E-14 |
| rs13143677 | A | G | 0.2961 | 0.1667 | 0.0265 | 3.12E-10 |
| rs13162174 | T | G | 0.601 | -0.1428 | 0.0246 | 6.45E-09 |
| rs1317507 | C | A | 0.7412 | -0.274 | 0.0277 | 3.74E-23 |
| rs13179413 | T | C | 0.2801 | 0.218 | 0.028 | 6.32E-15 |
| rs13206405 | A | C | 0.2 | 0.1776 | 0.03 | 3.34E-09 |
| rs13207962 | A | G | 0.2397 | -0.2242 | 0.0284 | 3.07E-15 |
| rs13216134 | G | A | 0.1079 | 0.3 | 0.0394 | 2.58E-14 |
| rs13234407 | A | G | 0.4867 | -0.2282 | 0.0239 | 1.39E-21 |
| rs1327235 | G | A | 0.4689 | 0.3732 | 0.0239 | 5.73E-55 |
| rs1327264 | C | A | 0.4402 | -0.1355 | 0.0242 | 2.24E-08 |
| rs1328360 | T | A | 0.3152 | -0.1871 | 0.0266 | 1.88E-12 |
| rs13328893 | T | C | 0.1545 | -0.3661 | 0.0353 | 3.54E-25 |
| rs13362497 | A | G | 0.0327 | -0.4838 | 0.0819 | 3.45E-09 |
| rs13399189 | A | T | 0.2898 | -0.2077 | 0.0269 | 1.20E-14 |
| rs13431652 | C | T | 0.3106 | -0.1493 | 0.0265 | 1.79E-08 |
| rs13432340 | G | A | 0.5657 | -0.1449 | 0.0244 | 2.73E-09 |
| rs1393218 | T | C | 0.5487 | 0.1493 | 0.024 | 5.17E-10 |
| rs1402166 | T | C | 0.269 | -0.1584 | 0.0273 | 6.62E-09 |
| rs140511912 | G | A | 0.6679 | -0.152 | 0.0258 | 3.60E-09 |
| rs1408945 | T | G | 0.4228 | -0.2818 | 0.0243 | 4.16E-31 |
| rs141212865 | C | A | 0.199 | -0.2664 | 0.0307 | 4.37E-18 |
| rs1415287 | T | C | 0.2896 | -0.1796 | 0.0264 | 1.05E-11 |
| rs141660869 | C | A | 0.0306 | -0.4763 | 0.0732 | 7.67E-11 |
| rs1421002 | C | T | 0.4736 | 0.1548 | 0.0243 | 2.00E-10 |
| rs142449193 | T | C | 0.046 | -0.3773 | 0.0604 | 4.09E-10 |
| rs143167197 | G | A | 0.072 | 0.3931 | 0.0492 | 1.43E-15 |
| rs1432564 | T | C | 0.564 | -0.1643 | 0.0242 | 1.15E-11 |
| rs1436138 | G | A | 0.3669 | -0.2478 | 0.0251 | 6.39E-23 |
| rs1440371 | A | G | 0.288 | 0.2106 | 0.0265 | 2.14E-15 |
| rs1452662 | T | C | 0.1014 | -0.355 | 0.0402 | 1.09E-18 |
| rs145781730 | C | G | 0.0436 | 0.3351 | 0.0596 | 1.87E-08 |
| rs1474698 | T | C | 0.5503 | -0.1934 | 0.0243 | 1.59E-15 |
| rs1479614 | A | G | 0.6039 | -0.1906 | 0.0249 | 2.16E-14 |
| rs149576225 | T | A | 0.1162 | 0.2325 | 0.0413 | 1.80E-08 |
| rs150857355 | C | G | 0.0217 | 0.8301 | 0.0895 | 1.71E-20 |
| rs1527351 | C | T | 0.3373 | -0.1811 | 0.0255 | 1.20E-12 |
| rs1527575 | G | A | 0.6414 | -0.1535 | 0.0253 | 1.38E-09 |
| rs1532717 | A | G | 0.5273 | -0.1605 | 0.0241 | 2.69E-11 |
| rs1543310 | C | T | 0.5408 | 0.1898 | 0.0252 | 4.69E-14 |
| rs1544861 | C | T | 0.6626 | -0.1851 | 0.0255 | 4.08E-13 |
| rs155522 | T | G | 0.5905 | -0.2027 | 0.0246 | 1.61E-16 |
| rs1558703 | T | A | 0.2378 | 0.1663 | 0.0282 | 3.57E-09 |
| rs1565715 | T | C | 0.0729 | 0.2626 | 0.0461 | 1.26E-08 |
| rs160838 | A | G | 0.5844 | 0.2972 | 0.0319 | 1.11E-20 |
| rs1621 | A | G | 0.6685 | -0.1696 | 0.0257 | 4.36E-11 |
| rs163049 | A | G | 0.6043 | 0.1568 | 0.0249 | 3.11E-10 |
| rs1630736 | T | C | 0.4622 | -0.1664 | 0.0246 | 1.39E-11 |
| rs1636608 | A | C | 0.7589 | 0.1672 | 0.0282 | 3.17E-09 |
| rs1642294 | G | C | 0.8593 | -0.2079 | 0.035 | 2.93E-09 |
| rs16892850 | C | T | 0.2756 | 0.1631 | 0.0273 | 2.39E-09 |
| rs16931177 | G | C | 0.6049 | -0.1909 | 0.0247 | 1.22E-14 |
| rs16939357 | C | T | 0.158 | -0.2717 | 0.0334 | 4.44E-16 |
| rs1694068 | A | T | 0.6161 | 0.2463 | 0.0248 | 3.28E-23 |
| rs17035181 | G | T | 0.1456 | -0.2949 | 0.0342 | 7.22E-18 |
| rs17035646 | A | G | 0.342 | 0.4588 | 0.0256 | 8.18E-72 |
| rs1709345 | G | T | 0.4596 | -0.1452 | 0.0243 | 2.30E-09 |
| rs17099316 | C | T | 0.0631 | -0.3108 | 0.0523 | 2.86E-09 |
| rs17115145 | T | C | 0.396 | 0.144 | 0.0247 | 5.92E-09 |
| rs17171710 | T | C | 0.1049 | -0.3994 | 0.0398 | 1.20E-23 |
| rs17189980 | C | T | 0.0371 | 0.4136 | 0.0663 | 4.37E-10 |
| rs17245822 | C | A | 0.3685 | 0.1456 | 0.0254 | 1.04E-08 |
| rs17400834 | C | T | 0.1428 | 0.1905 | 0.0347 | 3.83E-08 |
| rs1745417 | T | C | 0.5196 | 0.1834 | 0.0243 | 4.76E-14 |
| rs17474056 | T | C | 0.5627 | -0.2152 | 0.0243 | 9.23E-19 |
| rs17517959 | C | T | 0.3793 | 0.1564 | 0.0254 | 6.87E-10 |
| rs17543181 | A | G | 0.27 | 0.1605 | 0.0276 | 5.76E-09 |
| rs17562391 | T | C | 0.418 | 0.2067 | 0.0244 | 2.31E-17 |
| rs17608766 | C | T | 0.1403 | 0.5723 | 0.0348 | 7.46E-61 |
| rs17609994 | G | A | 0.2002 | 0.3069 | 0.0311 | 5.78E-23 |
| rs1764975 | A | T | 0.7992 | 0.2353 | 0.0303 | 8.71E-15 |
| rs17677603 | G | A | 0.383 | 0.3128 | 0.0248 | 2.36E-36 |
| rs17679259 | G | A | 0.1331 | -0.3583 | 0.0356 | 7.17E-24 |
| rs17684859 | C | T | 0.2643 | 0.1963 | 0.0271 | 4.72E-13 |
| rs17696741 | A | G | 0.1224 | 0.2716 | 0.0378 | 6.31E-13 |
| rs17706790 | A | G | 0.1741 | 0.2687 | 0.0322 | 7.60E-17 |
| rs17712705 | G | A | 0.6702 | -0.1949 | 0.0256 | 2.55E-14 |
| rs1779240 | A | G | 0.7662 | -0.3861 | 0.0285 | 7.24E-42 |
| rs17812022 | T | C | 0.0954 | -0.3153 | 0.043 | 2.26E-13 |
| rs1797912 | C | A | 0.3623 | -0.1658 | 0.0254 | 6.16E-11 |
| rs179972 | T | C | 0.4302 | 0.1407 | 0.0256 | 3.81E-08 |
| rs1801253 | C | G | 0.732 | 0.399 | 0.0278 | 9.76E-47 |
| rs182050989 | T | C | 0.0343 | 0.5292 | 0.0708 | 7.56E-14 |
| rs1852922 | A | G | 0.6845 | 0.1911 | 0.0262 | 3.31E-13 |
| rs1871190 | T | G | 0.3327 | 0.1444 | 0.0262 | 3.68E-08 |
| rs1886220 | G | A | 0.7556 | 0.204 | 0.0282 | 4.88E-13 |
| rs1898165 | A | G | 0.5247 | -0.1939 | 0.0241 | 8.22E-16 |
| rs190533862 | A | T | 0.0641 | 0.2875 | 0.0517 | 2.75E-08 |
| rs1906672 | A | G | 0.2323 | 0.2515 | 0.0286 | 1.44E-18 |
| rs1947461 | G | A | 0.3643 | 0.1428 | 0.0252 | 1.46E-08 |
| rs1962648 | T | C | 0.4198 | 0.1859 | 0.0244 | 2.51E-14 |
| rs1976450 | T | G | 0.1628 | -0.1941 | 0.0326 | 2.68E-09 |
| rs1983578 | A | C | 0.3956 | 0.1384 | 0.0248 | 2.27E-08 |
| rs1984195 | A | G | 0.4883 | 0.235 | 0.0244 | 5.68E-22 |
| rs198663 | C | T | 0.5292 | -0.1692 | 0.0247 | 8.15E-12 |
| rs198851 | G | T | 0.851 | -0.4454 | 0.0338 | 1.33E-39 |
| rs1995496 | G | A | 0.501 | 0.1637 | 0.0247 | 3.24E-11 |
| rs1998107 | G | A | 0.4975 | 0.1565 | 0.0241 | 9.15E-11 |
| rs2003931 | A | G | 0.3493 | -0.2124 | 0.0254 | 5.68E-17 |
| rs2011603 | A | G | 0.7365 | 0.2464 | 0.0275 | 3.14E-19 |
| rs2023844 | A | G | 0.9245 | 0.8082 | 0.0457 | 7.34E-70 |
| rs2042364 | G | A | 0.5358 | 0.1524 | 0.0241 | 2.80E-10 |
| rs2069845 | A | G | 0.5518 | -0.1913 | 0.0289 | 3.35E-11 |
| rs2071287 | T | C | 0.4828 | 0.2456 | 0.0248 | 4.77E-23 |
| rs2072927 | A | G | 0.4877 | -0.3138 | 0.0297 | 4.30E-26 |
| rs2075865 | A | C | 0.4086 | -0.1489 | 0.0246 | 1.47E-09 |
| rs2092867 | A | C | 0.6472 | 0.1452 | 0.0251 | 7.40E-09 |
| rs2093324 | A | G | 0.3736 | -0.1496 | 0.0252 | 2.97E-09 |
| rs210381 | A | G | 0.5619 | -0.1717 | 0.0245 | 2.57E-12 |
| rs2107595 | A | G | 0.1636 | 0.4262 | 0.0333 | 1.35E-37 |
| rs211307 | G | A | 0.2462 | 0.1782 | 0.028 | 1.87E-10 |
| rs2156805 | G | A | 0.5117 | -0.172 | 0.0242 | 1.20E-12 |
| rs2160236 | C | G | 0.3846 | -0.231 | 0.0258 | 2.94E-19 |
| rs2161967 | G | T | 0.5712 | -0.25 | 0.0246 | 2.89E-24 |
| rs2165197 | C | T | 0.4895 | 0.1833 | 0.0241 | 2.99E-14 |
| rs2178895 | T | C | 0.6391 | 0.1755 | 0.0252 | 3.65E-12 |
| rs2180052 | A | G | 0.1522 | -0.2126 | 0.0344 | 6.49E-10 |
| rs220249 | A | G | 0.4475 | 0.1599 | 0.0243 | 4.47E-11 |
| rs2208589 | G | A | 0.7745 | 0.2681 | 0.0302 | 6.29E-19 |
| rs2224858 | G | A | 0.815 | 0.1789 | 0.0309 | 7.37E-09 |
| rs2232460 | A | G | 0.335 | -0.1788 | 0.0258 | 4.32E-12 |
| rs2236295 | T | G | 0.3942 | -0.2203 | 0.0249 | 8.47E-19 |
| rs2236796 | C | G | 0.1604 | -0.7974 | 0.0327 | 4.29E-131 |
| rs2238787 | A | G | 0.2901 | 0.2381 | 0.0266 | 4.12E-19 |
| rs2243955 | G | A | 0.6361 | -0.1826 | 0.0255 | 7.88E-13 |
| rs2273171 | C | T | 0.4721 | 0.1347 | 0.0239 | 1.74E-08 |
| rs2279470 | G | C | 0.2155 | -0.1719 | 0.0295 | 5.55E-09 |
| rs2286526 | T | C | 0.7105 | 0.3706 | 0.0277 | 7.53E-41 |
| rs2288378 | C | T | 0.7527 | 0.2091 | 0.028 | 7.99E-14 |
| rs2289124 | A | G | 0.1683 | -0.2968 | 0.0337 | 1.41E-18 |
| rs2290263 | A | G | 0.7547 | 0.2014 | 0.0281 | 7.35E-13 |
| rs2291433 | G | T | 0.5348 | -0.2327 | 0.0242 | 7.13E-22 |
| rs2297603 | T | G | 0.1086 | 0.2387 | 0.0395 | 1.48E-09 |
| rs2298359 | C | T | 0.0622 | -0.4544 | 0.0668 | 1.00E-11 |
| rs2300481 | T | C | 0.3847 | 0.1924 | 0.0248 | 8.16E-15 |
| rs2304130 | G | A | 0.0841 | 0.3065 | 0.044 | 3.22E-12 |
| rs2306363 | T | G | 0.2013 | -0.4046 | 0.0301 | 4.44E-41 |
| rs2315518 | C | G | 0.3377 | 0.1467 | 0.0253 | 6.99E-09 |
| rs2328813 | A | C | 0.5734 | -0.1453 | 0.0244 | 2.67E-09 |
| rs234623 | A | G | 0.5067 | -0.1382 | 0.0243 | 1.23E-08 |
| rs2354862 | C | A | 0.3576 | -0.1798 | 0.0253 | 1.09E-12 |
| rs2367112 | G | T | 0.4953 | 0.1354 | 0.0241 | 1.88E-08 |
| rs2368566 | A | G | 0.92 | 0.3284 | 0.0576 | 1.20E-08 |
| rs2379829 | C | G | 0.7247 | -0.2087 | 0.0277 | 5.13E-14 |
| rs2389873 | G | A | 0.4814 | 0.1468 | 0.0243 | 1.58E-09 |
| rs2392929 | G | T | 0.2021 | 0.7111 | 0.0301 | 1.59E-123 |
| rs2393455 | A | C | 0.5673 | 0.1475 | 0.0247 | 2.45E-09 |
| rs2395622 | T | C | 0.8158 | -0.186 | 0.0311 | 2.22E-09 |
| rs2419077 | T | G | 0.2607 | 0.1755 | 0.0276 | 1.97E-10 |
| rs2422074 | C | T | 0.2165 | 0.167 | 0.0291 | 9.74E-09 |
| rs2423514 | G | A | 0.4572 | -0.2601 | 0.0243 | 7.99E-27 |
| rs243033 | C | G | 0.2871 | 0.1489 | 0.0268 | 2.84E-08 |
| rs2493296 | T | C | 0.1437 | 0.3286 | 0.0348 | 4.12E-21 |
| rs2494180 | C | T | 0.5732 | 0.1711 | 0.0244 | 2.54E-12 |
| rs2497311 | C | T | 0.8977 | -0.2903 | 0.0475 | 1.01E-09 |
| rs2529053 | G | A | 0.1794 | -0.1931 | 0.0318 | 1.22E-09 |
| rs2540949 | T | A | 0.3805 | -0.2606 | 0.0248 | 9.71E-26 |
| rs2569882 | C | T | 0.4349 | -0.1982 | 0.0251 | 2.66E-15 |
| rs2574985 | G | A | 0.718 | 0.1493 | 0.0269 | 2.80E-08 |
| rs2577024 | G | A | 0.2086 | 0.2015 | 0.0296 | 9.54E-12 |
| rs2608029 | G | C | 0.3398 | -0.1507 | 0.0258 | 5.35E-09 |
| rs2611774 | A | G | 0.3371 | 0.2147 | 0.0257 | 6.45E-17 |
| rs2613765 | A | G | 0.4698 | -0.2228 | 0.0241 | 2.02E-20 |
| rs2627316 | G | A | 0.4684 | 0.3106 | 0.0241 | 5.53E-38 |
| rs263017 | G | A | 0.508 | -0.1907 | 0.0241 | 2.60E-15 |
| rs263533 | T | C | 0.4202 | -0.1606 | 0.0246 | 6.71E-11 |
| rs2643826 | T | C | 0.4522 | 0.4177 | 0.0247 | 5.54E-64 |
| rs2744133 | G | A | 0.2742 | -0.2621 | 0.0271 | 3.65E-22 |
| rs2753960 | T | G | 0.424 | 0.3651 | 0.0245 | 2.04E-50 |
| rs278123 | A | G | 0.3179 | 0.1418 | 0.0259 | 4.37E-08 |
| rs2801008 | G | T | 0.3191 | 0.1757 | 0.0262 | 2.01E-11 |
| rs2807337 | C | T | 0.63 | -0.1718 | 0.0249 | 4.91E-12 |
| rs2815063 | A | C | 0.1314 | 0.232 | 0.0367 | 2.75E-10 |
| rs2823139 | A | G | 0.3386 | 0.2282 | 0.0259 | 1.33E-18 |
| rs2833834 | A | C | 0.2797 | 0.1908 | 0.027 | 1.63E-12 |
| rs28558845 | C | G | 0.1556 | -0.2006 | 0.0335 | 2.14E-09 |
| rs28578714 | C | T | 0.3918 | -0.1844 | 0.0255 | 4.71E-13 |
| rs28590346 | T | A | 0.3405 | 0.2605 | 0.0261 | 1.59E-23 |
| rs28601620 | T | C | 0.1974 | 0.2789 | 0.0305 | 5.27E-20 |
| rs28611491 | T | C | 0.0876 | 0.2456 | 0.045 | 4.96E-08 |
| rs2872717 | C | T | 0.2444 | 0.2266 | 0.0281 | 7.95E-16 |
| rs28786478 | T | C | 0.1068 | 0.2441 | 0.0389 | 3.44E-10 |
| rs289398 | G | A | 0.8495 | -0.1964 | 0.0338 | 6.06E-09 |
| rs2906157 | G | A | 0.6421 | -0.237 | 0.0255 | 1.72E-20 |
| rs2920926 | G | A | 0.7306 | 0.259 | 0.0276 | 6.36E-21 |
| rs2935462 | A | C | 0.8 | -0.1743 | 0.0301 | 7.29E-09 |
| rs2943646 | G | A | 0.6433 | 0.2709 | 0.025 | 1.94E-27 |
| rs2948094 | A | G | 0.3846 | -0.2011 | 0.0248 | 4.89E-16 |
| rs296797 | C | T | 0.5954 | -0.1529 | 0.0245 | 4.66E-10 |
| rs2978098 | C | A | 0.4584 | -0.1934 | 0.0243 | 1.67E-15 |
| rs2978398 | A | G | 0.4228 | -0.1522 | 0.0248 | 9.06E-10 |
| rs2978456 | C | T | 0.4488 | 0.1764 | 0.0257 | 7.07E-12 |
| rs2979470 | C | T | 0.5128 | -0.1549 | 0.0247 | 3.55E-10 |
| rs2997336 | A | G | 0.5349 | -0.1343 | 0.0242 | 2.71E-08 |
| rs3098186 | T | C | 0.5174 | -0.202 | 0.0244 | 1.28E-16 |
| rs3110053 | G | C | 0.599 | -0.1795 | 0.0246 | 2.62E-13 |
| rs312023 | A | G | 0.535 | -0.1782 | 0.0247 | 5.48E-13 |
| rs3125001 | T | C | 0.3779 | 0.1602 | 0.0257 | 4.68E-10 |
| rs3129087 | T | C | 0.1789 | 0.1941 | 0.0319 | 1.18E-09 |
| rs33996239 | T | C | 0.0603 | -0.3175 | 0.0548 | 7.09E-09 |
| rs34087251 | C | T | 0.0576 | 0.3013 | 0.0519 | 6.50E-09 |
| rs34130368 | T | G | 0.117 | -0.2515 | 0.0387 | 7.87E-11 |
| rs34484182 | G | A | 0.5006 | 0.1431 | 0.024 | 2.67E-09 |
| rs34869093 | G | A | 0.3707 | 0.1758 | 0.0252 | 2.83E-12 |
| rs34905952 | A | G | 0.16 | 0.2401 | 0.0329 | 2.78E-13 |
| rs34991912 | C | T | 0.575 | -0.2677 | 0.0246 | 1.31E-27 |
| rs35107212 | G | A | 0.4021 | 0.2168 | 0.0248 | 2.53E-18 |
| rs35123781 | G | A | 0.3407 | -0.1502 | 0.0256 | 4.78E-09 |
| rs35413927 | G | A | 0.3043 | 0.2462 | 0.0266 | 2.10E-20 |
| rs35429 | G | A | 0.3874 | -0.3911 | 0.0249 | 2.11E-55 |
| rs35450617 | G | T | 0.2988 | 0.1485 | 0.0266 | 2.47E-08 |
| rs35680304 | T | C | 0.5899 | 0.239 | 0.0248 | 5.86E-22 |
| rs356846 | C | G | 0.229 | -0.1754 | 0.0286 | 8.97E-10 |
| rs357489 | A | T | 0.7424 | -0.2229 | 0.0276 | 7.51E-16 |
| rs35761891 | G | A | 0.047 | 0.5631 | 0.0824 | 8.09E-12 |
| rs35783704 | A | G | 0.1009 | -0.4395 | 0.0417 | 6.07E-26 |
| rs35825793 | G | C | 0.3318 | 0.1483 | 0.0258 | 8.73E-09 |
| rs35973496 | T | C | 0.4407 | 0.4228 | 0.0242 | 2.67E-68 |
| rs36006409 | G | T | 0.2042 | 0.2009 | 0.0306 | 5.24E-11 |
| rs36563 | G | T | 0.8445 | 0.2062 | 0.0333 | 6.21E-10 |
| rs365990 | G | A | 0.3681 | -0.1717 | 0.0248 | 4.57E-12 |
| rs3740781 | T | C | 0.7259 | 0.2059 | 0.0276 | 7.98E-14 |
| rs3744010 | A | G | 0.2483 | -0.2456 | 0.028 | 1.80E-18 |
| rs3752440 | A | G | 0.0843 | 0.3282 | 0.0455 | 5.67E-13 |
| rs3754944 | A | C | 0.5856 | 0.1347 | 0.0245 | 3.97E-08 |
| rs37613 | C | T | 0.6707 | -0.1522 | 0.0256 | 2.84E-09 |
| rs3772219 | C | A | 0.3162 | -0.2457 | 0.0258 | 1.76E-21 |
| rs3786516 | A | T | 0.7767 | 0.2 | 0.0291 | 6.12E-12 |
| rs3796584 | A | G | 0.3623 | -0.2753 | 0.0249 | 2.28E-28 |
| rs3802228 | G | A | 0.5447 | -0.2455 | 0.0242 | 4.09E-24 |
| rs3807925 | G | A | 0.3497 | 0.1475 | 0.0254 | 6.21E-09 |
| rs3811640 | A | C | 0.2783 | -0.2005 | 0.0268 | 8.05E-14 |
| rs3815460 | G | C | 0.1032 | 0.2287 | 0.0396 | 7.51E-09 |
| rs3821843 | A | G | 0.6828 | 0.3071 | 0.027 | 6.29E-30 |
| rs3825568 | T | C | 0.4613 | -0.14 | 0.0241 | 5.95E-09 |
| rs3843712 | C | T | 0.1014 | 0.322 | 0.0477 | 1.50E-11 |
| rs3867466 | C | A | 0.2836 | 0.1618 | 0.0269 | 1.73E-09 |
| rs3915499 | A | G | 0.3191 | -0.1802 | 0.0258 | 3.15E-12 |
| rs3918226 | T | C | 0.0804 | 0.5655 | 0.0467 | 8.94E-34 |
| rs3935505 | G | T | 0.2509 | 0.155 | 0.028 | 3.19E-08 |
| rs40270 | C | A | 0.7677 | 0.2008 | 0.0288 | 2.93E-12 |
| rs42032 | A | G | 0.2658 | -0.2964 | 0.0273 | 1.88E-27 |
| rs4245930 | A | G | 0.6331 | -0.1956 | 0.025 | 5.56E-15 |
| rs4260863 | G | C | 0.39 | -0.1878 | 0.025 | 5.36E-14 |
| rs4298914 | T | C | 0.4577 | -0.1385 | 0.0242 | 9.86E-09 |
| rs4319878 | T | C | 0.5615 | 0.1356 | 0.0246 | 3.61E-08 |
| rs4320727 | A | G | 0.63 | 0.1804 | 0.0253 | 9.55E-13 |
| rs4374967 | G | A | 0.59 | -0.1518 | 0.0244 | 5.33E-10 |
| rs440597 | C | T | 0.2822 | 0.1888 | 0.0271 | 3.02E-12 |
| rs4414647 | G | A | 0.3942 | 0.1377 | 0.025 | 3.44E-08 |
| rs4478172 | C | A | 0.2574 | -0.178 | 0.0278 | 1.55E-10 |
| rs4489092 | T | G | 0.6305 | -0.1411 | 0.0254 | 2.81E-08 |
| rs4499010 | T | G | 0.6624 | 0.1507 | 0.0254 | 2.95E-09 |
| rs4506565 | T | A | 0.3146 | 0.2257 | 0.0259 | 3.20E-18 |
| rs4511593 | T | C | 0.6518 | -0.2584 | 0.0255 | 3.96E-24 |
| rs453528 | C | A | 0.4794 | -0.2098 | 0.0254 | 1.37E-16 |
| rs4573493 | C | T | 0.4907 | -0.1381 | 0.0243 | 1.42E-08 |
| rs4577304 | C | T | 0.4767 | 0.1375 | 0.0242 | 1.40E-08 |
| rs4590447 | G | A | 0.096 | 0.2768 | 0.0413 | 2.03E-11 |
| rs4599529 | A | G | 0.5073 | -0.1526 | 0.0243 | 3.62E-10 |
| rs4637011 | C | G | 0.306 | -0.2949 | 0.0266 | 1.24E-28 |
| rs464462 | C | T | 0.786 | 0.1662 | 0.0295 | 1.76E-08 |
| rs4651224 | T | C | 0.4496 | 0.1914 | 0.0247 | 9.25E-15 |
| rs4667454 | G | A | 0.3257 | -0.2178 | 0.0259 | 3.84E-17 |
| rs4675682 | C | T | 0.4563 | 0.2421 | 0.0245 | 4.21E-23 |
| rs4681161 | C | T | 0.5632 | 0.1342 | 0.0243 | 3.48E-08 |
| rs4691666 | T | C | 0.5325 | -0.2843 | 0.0243 | 1.05E-31 |
| rs4702102 | T | A | 0.6983 | -0.1805 | 0.0329 | 4.02E-08 |
| rs4713650 | T | C | 0.6322 | -0.1411 | 0.0252 | 2.08E-08 |
| rs4737371 | A | G | 0.1915 | -0.1879 | 0.0314 | 2.06E-09 |
| rs4739832 | C | A | 0.4118 | -0.1946 | 0.0247 | 3.04E-15 |
| rs4746139 | C | A | 0.1417 | 0.4099 | 0.0349 | 7.00E-32 |
| rs4754196 | G | A | 0.4758 | 0.2865 | 0.0242 | 2.15E-32 |
| rs4763999 | G | A | 0.5671 | -0.1523 | 0.0247 | 6.94E-10 |
| rs4767332 | A | C | 0.5725 | 0.2184 | 0.0241 | 1.53E-19 |
| rs4771653 | T | C | 0.6772 | -0.1577 | 0.0258 | 9.50E-10 |
| rs4790309 | T | C | 0.4487 | -0.2751 | 0.0244 | 1.92E-29 |
| rs4793069 | A | G | 0.9402 | 0.2954 | 0.0534 | 3.16E-08 |
| rs4795637 | C | T | 0.2354 | 0.1869 | 0.0283 | 4.30E-11 |
| rs4812536 | G | A | 0.3422 | -0.2155 | 0.0258 | 6.53E-17 |
| rs4831182 | A | G | 0.4985 | -0.1687 | 0.0242 | 3.08E-12 |
| rs4838021 | T | C | 0.13 | -0.2421 | 0.0365 | 3.16E-11 |
| rs4854511 | C | A | 0.3679 | 0.163 | 0.0253 | 1.22E-10 |
| rs4854572 | A | G | 0.4557 | -0.1664 | 0.0245 | 1.04E-11 |
| rs4859681 | A | C | 0.5296 | -0.2064 | 0.0241 | 1.02E-17 |
| rs4871964 | G | A | 0.2508 | -0.3124 | 0.0279 | 3.63E-29 |
| rs4888408 | A | G | 0.5851 | 0.3305 | 0.0245 | 1.70E-41 |
| rs4890500 | G | A | 0.2983 | 0.2383 | 0.0262 | 8.39E-20 |
| rs4918065 | C | T | 0.2492 | -0.2424 | 0.0281 | 5.82E-18 |
| rs4925159 | A | G | 0.4252 | 0.2184 | 0.0243 | 2.71E-19 |
| rs4926499 | C | G | 0.8245 | 0.2955 | 0.0368 | 9.46E-16 |
| rs4938353 | A | G | 0.8235 | -0.2864 | 0.0318 | 2.07E-19 |
| rs4948099 | A | C | 0.7504 | -0.1583 | 0.0284 | 2.61E-08 |
| rs4954192 | T | C | 0.391 | -0.1797 | 0.0312 | 8.66E-09 |
| rs4956929 | A | G | 0.5092 | -0.1564 | 0.0252 | 5.45E-10 |
| rs4961230 | A | G | 0.299 | 0.2034 | 0.0269 | 3.78E-14 |
| rs4963739 | C | T | 0.5003 | -0.1468 | 0.0242 | 1.24E-09 |
| rs4965529 | A | C | 0.1694 | -0.2519 | 0.0323 | 5.89E-15 |
| rs4980515 | C | T | 0.5046 | -0.1934 | 0.0247 | 4.90E-15 |
| rs5030779 | T | C | 0.2031 | -0.1781 | 0.0312 | 1.16E-08 |
| rs512730 | A | T | 0.194 | 0.2124 | 0.0304 | 3.01E-12 |
| rs532093 | A | G | 0.2096 | 0.2059 | 0.0308 | 2.18E-11 |
| rs538180 | A | T | 0.4167 | -0.1506 | 0.0245 | 8.47E-10 |
| rs551510 | T | C | 0.5037 | 0.1378 | 0.0244 | 1.65E-08 |
| rs55709426 | A | G | 0.2396 | 0.1919 | 0.0287 | 2.13E-11 |
| rs55828241 | G | C | 0.0577 | 0.3141 | 0.0518 | 1.35E-09 |
| rs55829517 | C | G | 0.117 | -0.3603 | 0.0374 | 5.51E-22 |
| rs55881012 | C | A | 0.4993 | 0.1939 | 0.0241 | 7.89E-16 |
| rs55925664 | A | T | 0.1885 | 0.4119 | 0.031 | 3.31E-40 |
| rs55938136 | G | A | 0.2447 | -0.2515 | 0.0389 | 1.05E-10 |
| rs56054109 | A | G | 0.4932 | -0.1356 | 0.0246 | 3.50E-08 |
| rs56083529 | A | C | 0.1253 | 0.2057 | 0.0365 | 1.81E-08 |
| rs56085433 | A | G | 0.1448 | -0.2219 | 0.0355 | 4.10E-10 |
| rs56213443 | A | C | 0.5312 | -0.1787 | 0.025 | 8.50E-13 |
| rs56350535 | A | G | 0.1232 | -0.2071 | 0.0377 | 3.85E-08 |
| rs56352451 | T | C | 0.1328 | 0.2009 | 0.0353 | 1.31E-08 |
| rs56373125 | G | A | 0.0621 | -0.3085 | 0.0533 | 7.32E-09 |
| rs56388530 | T | C | 0.7613 | 0.3744 | 0.0283 | 6.92E-40 |
| rs56389811 | T | C | 0.2311 | -0.245 | 0.0286 | 9.42E-18 |
| rs568032 | G | A | 0.9411 | 0.3775 | 0.0538 | 2.19E-12 |
| rs569550 | G | T | 0.3964 | 0.4891 | 0.0253 | 2.01E-83 |
| rs57062879 | G | A | 0.5196 | -0.1374 | 0.0241 | 1.13E-08 |
| rs57140819 | G | C | 0.173 | -0.2022 | 0.0321 | 2.91E-10 |
| rs572428 | A | G | 0.3303 | 0.1779 | 0.0257 | 4.83E-12 |
| rs572618 | T | G | 0.325 | -0.1395 | 0.0256 | 4.80E-08 |
| rs57400569 | A | G | 0.2216 | -0.2434 | 0.0291 | 6.01E-17 |
| rs57541197 | A | G | 0.14 | -0.5336 | 0.0346 | 1.39E-53 |
| rs5756813 | T | G | 0.6129 | -0.1506 | 0.025 | 1.81E-09 |
| rs57748895 | T | A | 0.0186 | 0.7931 | 0.0921 | 7.00E-18 |
| rs57851860 | C | T | 0.6222 | -0.1737 | 0.0249 | 2.93E-12 |
| rs57917116 | G | A | 0.0963 | 0.2666 | 0.0407 | 6.00E-11 |
| rs58413618 | C | T | 0.3308 | 0.1786 | 0.0258 | 4.28E-12 |
| rs588177 | A | C | 0.6834 | -0.1651 | 0.0259 | 1.91E-10 |
| rs592440 | A | T | 0.6859 | 0.1646 | 0.0258 | 1.93E-10 |
| rs594613 | C | G | 0.8629 | -0.2543 | 0.0354 | 6.88E-13 |
| rs59484271 | T | C | 0.2133 | 0.2024 | 0.0297 | 8.97E-12 |
| rs597808 | G | A | 0.5094 | -0.5614 | 0.0292 | 1.57E-82 |
| rs5992134 | T | G | 0.2383 | 0.1557 | 0.0285 | 4.69E-08 |
| rs5997409 | C | T | 0.1564 | 0.2679 | 0.0332 | 6.56E-16 |
| rs60121503 | C | G | 0.0843 | -0.2384 | 0.0433 | 3.68E-08 |
| rs601338 | A | G | 0.491 | 0.1765 | 0.0241 | 2.49E-13 |
| rs60171845 | C | A | 0.1816 | 0.1835 | 0.0314 | 4.93E-09 |
| rs6017279 | C | T | 0.1345 | 0.3864 | 0.0351 | 3.85E-28 |
| rs6021247 | A | G | 0.5326 | 0.2046 | 0.024 | 1.40E-17 |
| rs6026739 | T | A | 0.1236 | 0.562 | 0.0369 | 2.27E-52 |
| rs6039211 | G | A | 0.3659 | -0.1808 | 0.025 | 5.07E-13 |
| rs604723 | C | T | 0.7223 | 0.5377 | 0.0273 | 2.12E-86 |
| rs6062344 | T | C | 0.4969 | -0.2631 | 0.0245 | 7.76E-27 |
| rs6078093 | A | G | 0.4244 | -0.1738 | 0.0243 | 8.77E-13 |
| rs6085527 | G | A | 0.5938 | 0.195 | 0.0246 | 2.44E-15 |
| rs6088749 | G | C | 0.165 | -0.2376 | 0.0324 | 2.22E-13 |
| rs60909079 | C | G | 0.2492 | -0.1998 | 0.0279 | 8.02E-13 |
| rs61148001 | T | C | 0.2159 | -0.2151 | 0.0296 | 3.73E-13 |
| rs61384251 | G | A | 0.1056 | 0.2287 | 0.0398 | 9.22E-09 |
| rs61772592 | G | A | 0.1241 | 0.2693 | 0.037 | 3.29E-13 |
| rs61789367 | A | G | 0.0451 | 0.3355 | 0.0599 | 2.10E-08 |
| rs61874877 | A | G | 0.2992 | 0.1884 | 0.0266 | 1.46E-12 |
| rs61911503 | A | G | 0.1225 | 0.2214 | 0.0375 | 3.44E-09 |
| rs61915422 | G | C | 0.129 | -0.2034 | 0.0369 | 3.55E-08 |
| rs61942600 | G | A | 0.0823 | 0.2548 | 0.0456 | 2.23E-08 |
| rs62055044 | T | G | 0.047 | 0.4405 | 0.0582 | 3.83E-14 |
| rs62055084 | T | C | 0.2699 | 0.2174 | 0.0283 | 1.55E-14 |
| rs62082230 | A | T | 0.2779 | -0.1656 | 0.0277 | 2.38E-09 |
| rs62158170 | G | A | 0.2167 | -0.1933 | 0.0295 | 6.11E-11 |
| rs62179715 | T | C | 0.2959 | -0.2444 | 0.0264 | 2.22E-20 |
| rs62189054 | G | C | 0.1013 | -0.238 | 0.0405 | 4.12E-09 |
| rs62229372 | T | C | 0.1237 | 0.2177 | 0.0389 | 2.19E-08 |
| rs62271373 | A | T | 0.0592 | 0.4389 | 0.0539 | 4.06E-16 |
| rs62301873 | G | A | 0.1054 | 0.2203 | 0.0394 | 2.29E-08 |
| rs62361304 | T | C | 0.1591 | -0.2001 | 0.0329 | 1.26E-09 |
| rs62434125 | C | T | 0.0691 | -0.6935 | 0.0475 | 2.65E-48 |
| rs62445442 | A | G | 0.0278 | 0.4386 | 0.0754 | 6.12E-09 |
| rs62477685 | T | A | 0.4116 | -0.1587 | 0.0265 | 1.99E-09 |
| rs62480403 | G | C | 0.3177 | -0.1943 | 0.0262 | 1.21E-13 |
| rs6271 | T | C | 0.072 | -0.4579 | 0.0489 | 8.24E-21 |
| rs629864 | T | C | 0.6524 | -0.1621 | 0.0259 | 3.68E-10 |
| rs640935 | C | T | 0.2396 | 0.1724 | 0.0283 | 1.13E-09 |
| rs6434200 | C | T | 0.721 | -0.1739 | 0.027 | 1.24E-10 |
| rs6438857 | C | T | 0.4252 | -0.2145 | 0.0245 | 2.29E-18 |
| rs6445054 | C | T | 0.1719 | 0.1926 | 0.0323 | 2.46E-09 |
| rs6452769 | A | G | 0.2116 | -0.2179 | 0.0297 | 2.34E-13 |
| rs6504023 | T | C | 0.7695 | -0.2053 | 0.0287 | 8.91E-13 |
| rs6504213 | C | T | 0.5844 | 0.2417 | 0.0251 | 6.25E-22 |
| rs6517423 | A | G | 0.2957 | 0.1478 | 0.0264 | 2.17E-08 |
| rs6544684 | C | T | 0.6262 | -0.17 | 0.0248 | 7.76E-12 |
| rs6558537 | G | A | 0.7215 | 0.1931 | 0.0275 | 2.16E-12 |
| rs6560653 | G | A | 0.6879 | -0.149 | 0.026 | 1.03E-08 |
| rs6563493 | G | A | 0.322 | -0.2178 | 0.0257 | 2.54E-17 |
| rs6565174 | C | A | 0.8898 | 0.2655 | 0.0389 | 9.07E-12 |
| rs6678140 | T | C | 0.6672 | -0.1807 | 0.0254 | 1.21E-12 |
| rs66893799 | A | G | 0.0189 | 0.7042 | 0.0931 | 3.92E-14 |
| rs6714150 | G | A | 0.6315 | -0.2149 | 0.0253 | 2.17E-17 |
| rs6723509 | C | T | 0.141 | -0.2563 | 0.0346 | 1.37E-13 |
| rs6723772 | T | C | 0.1048 | -0.2272 | 0.0402 | 1.55E-08 |
| rs6729623 | G | A | 0.4957 | -0.141 | 0.0242 | 5.88E-09 |
| rs6731373 | A | G | 0.3451 | 0.1816 | 0.0263 | 4.64E-12 |
| rs6732308 | G | A | 0.2028 | -0.2877 | 0.0302 | 1.81E-21 |
| rs6734118 | A | C | 0.2167 | -0.3217 | 0.0347 | 1.87E-20 |
| rs6736786 | G | A | 0.4006 | 0.1706 | 0.0248 | 6.03E-12 |
| rs6742 | C | T | 0.7844 | -0.244 | 0.0319 | 2.14E-14 |
| rs67468157 | T | C | 0.3119 | 0.1571 | 0.026 | 1.51E-09 |
| rs67596972 | G | A | 0.3705 | -0.1835 | 0.0264 | 3.47E-12 |
| rs6771917 | C | T | 0.7472 | 0.32 | 0.0286 | 5.52E-29 |
| rs6777317 | A | G | 0.2955 | 0.1714 | 0.0268 | 1.71E-10 |
| rs6778977 | G | T | 0.3421 | 0.1797 | 0.0256 | 2.25E-12 |
| rs678214 | C | T | 0.3409 | -0.1427 | 0.0256 | 2.57E-08 |
| rs67822851 | G | A | 0.39 | -0.1434 | 0.0252 | 1.34E-08 |
| rs67909753 | A | G | 0.2894 | -0.2251 | 0.0266 | 2.44E-17 |
| rs6806529 | C | A | 0.5703 | -0.1495 | 0.0249 | 1.83E-09 |
| rs68080737 | A | G | 0.0656 | 0.2963 | 0.0489 | 1.42E-09 |
| rs6823767 | C | T | 0.2766 | 0.1663 | 0.0271 | 8.00E-10 |
| rs6832891 | C | T | 0.2717 | 0.1514 | 0.0276 | 4.04E-08 |
| rs687914 | T | G | 0.2531 | 0.2323 | 0.0284 | 3.12E-16 |
| rs688540 | A | G | 0.8667 | 0.2249 | 0.0365 | 6.96E-10 |
| rs6890251 | T | C | 0.1579 | 0.3641 | 0.0332 | 5.52E-28 |
| rs689304 | T | C | 0.0974 | -0.3961 | 0.0408 | 3.00E-22 |
| rs6897211 | C | T | 0.2621 | -0.1683 | 0.0277 | 1.16E-09 |
| rs6905288 | A | G | 0.5671 | 0.1641 | 0.0246 | 2.50E-11 |
| rs6921291 | T | C | 0.1906 | 0.3343 | 0.0316 | 3.99E-26 |
| rs6921610 | T | C | 0.5371 | -0.1616 | 0.0242 | 2.23E-11 |
| rs6926566 | T | C | 0.4277 | -0.1554 | 0.0242 | 1.45E-10 |
| rs6928692 | C | T | 0.4897 | 0.1623 | 0.0287 | 1.52E-08 |
| rs6932812 | G | C | 0.0701 | -0.3389 | 0.0485 | 2.86E-12 |
| rs6959688 | G | A | 0.4022 | 0.197 | 0.0249 | 2.80E-15 |
| rs6961048 | G | C | 0.1046 | 0.5082 | 0.0395 | 7.03E-38 |
| rs6979606 | T | C | 0.6195 | 0.1422 | 0.0253 | 1.90E-08 |
| rs6983239 | T | G | 0.2242 | 0.1641 | 0.0293 | 2.17E-08 |
| rs699 | G | A | 0.4117 | 0.327 | 0.0244 | 8.01E-41 |
| rs7003445 | C | T | 0.4088 | 0.162 | 0.0248 | 6.72E-11 |
| rs7006340 | G | A | 0.3054 | 0.1624 | 0.0264 | 7.19E-10 |
| rs7009170 | C | T | 0.6819 | 0.164 | 0.0259 | 2.47E-10 |
| rs7012866 | G | T | 0.5004 | 0.2012 | 0.024 | 5.38E-17 |
| rs7023828 | T | C | 0.4165 | -0.2538 | 0.0246 | 4.91E-25 |
| rs704191 | C | T | 0.5378 | -0.1415 | 0.0244 | 6.44E-09 |
| rs7045409 | A | T | 0.3705 | -0.1766 | 0.0252 | 2.25E-12 |
| rs706159 | A | G | 0.4301 | -0.1824 | 0.0247 | 1.70E-13 |
| rs7083519 | G | C | 0.6157 | -0.2011 | 0.0247 | 4.08E-16 |
| rs709668 | G | A | 0.7968 | 0.2584 | 0.0302 | 1.08E-17 |
| rs7099692 | C | G | 0.7118 | -0.1796 | 0.0268 | 2.21E-11 |
| rs7107202 | A | T | 0.8197 | 0.361 | 0.0345 | 1.24E-25 |
| rs7107356 | G | A | 0.5041 | 0.3874 | 0.024 | 2.08E-58 |
| rs7109016 | T | A | 0.704 | 0.1792 | 0.0263 | 9.55E-12 |
| rs7110547 | G | C | 0.3824 | 0.179 | 0.0251 | 1.03E-12 |
| rs7118562 | G | A | 0.1244 | -0.3288 | 0.0367 | 3.32E-19 |
| rs7125196 | C | T | 0.1181 | -0.3417 | 0.0374 | 7.19E-20 |
| rs714277 | T | C | 0.2813 | 0.1798 | 0.0266 | 1.44E-11 |
| rs715 | C | T | 0.3129 | -0.2085 | 0.0262 | 1.91E-15 |
| rs7160184 | T | C | 0.0944 | -0.241 | 0.0415 | 6.22E-09 |
| rs7174174 | C | T | 0.2259 | 0.1998 | 0.0292 | 8.02E-12 |
| rs7187540 | A | C | 0.3346 | -0.1621 | 0.0271 | 2.18E-09 |
| rs7200353 | A | G | 0.6107 | -0.1567 | 0.0252 | 4.95E-10 |
| rs7210475 | G | A | 0.6323 | -0.1632 | 0.0253 | 1.15E-10 |
| rs7210771 | A | C | 0.1419 | 0.2231 | 0.0348 | 1.51E-10 |
| rs7211535 | G | A | 0.5208 | 0.1411 | 0.0246 | 9.37E-09 |
| rs7213273 | A | G | 0.6562 | -0.3234 | 0.0252 | 1.42E-37 |
| rs7214135 | T | C | 0.0568 | 0.3094 | 0.0533 | 6.63E-09 |
| rs7236548 | A | C | 0.1856 | 0.2819 | 0.031 | 9.74E-20 |
| rs7246865 | A | G | 0.2708 | 0.2918 | 0.0277 | 5.16E-26 |
| rs7250497 | G | A | 0.3502 | -0.1681 | 0.0256 | 5.35E-11 |
| rs7255 | C | T | 0.5425 | 0.1895 | 0.0242 | 5.18E-15 |
| rs7255425 | A | G | 0.1945 | 0.1975 | 0.0341 | 7.15E-09 |
| rs726816 | G | T | 0.5333 | -0.1579 | 0.0242 | 6.55E-11 |
| rs72688070 | T | C | 0.1721 | -0.228 | 0.0322 | 1.33E-12 |
| rs72719160 | T | A | 0.3199 | 0.2288 | 0.0258 | 8.54E-19 |
| rs72756346 | T | G | 0.282 | -0.2061 | 0.0315 | 5.77E-11 |
| rs72793375 | A | G | 0.2163 | 0.18 | 0.0308 | 4.91E-09 |
| rs72803938 | C | A | 0.2488 | -0.1855 | 0.0283 | 5.90E-11 |
| rs72844527 | A | C | 0.1652 | 0.2051 | 0.0326 | 2.95E-10 |
| rs72910071 | A | G | 0.108 | 0.3753 | 0.0405 | 1.80E-20 |
| rs72914576 | G | C | 0.1908 | 0.2513 | 0.0307 | 2.91E-16 |
| rs72917789 | T | C | 0.0688 | -0.2768 | 0.0485 | 1.14E-08 |
| rs72931748 | G | A | 0.0946 | -0.3395 | 0.0426 | 1.48E-15 |
| rs72976750 | C | T | 0.1386 | 0.4688 | 0.0352 | 1.41E-40 |
| rs7302981 | G | A | 0.623 | -0.3553 | 0.0246 | 2.10E-47 |
| rs73033340 | G | A | 0.0302 | -0.7233 | 0.0897 | 7.56E-16 |
| rs73046792 | A | G | 0.1528 | -0.2912 | 0.0352 | 1.32E-16 |
| rs73049928 | G | A | 0.1913 | 0.1949 | 0.0315 | 6.08E-10 |
| rs7306710 | C | T | 0.5238 | 0.2095 | 0.0243 | 7.20E-18 |
| rs7307248 | T | C | 0.2494 | 0.1622 | 0.0284 | 1.13E-08 |
| rs73075659 | G | A | 0.3315 | -0.3431 | 0.0259 | 5.56E-40 |
| rs7315980 | A | G | 0.0822 | 0.4392 | 0.045 | 1.55E-22 |
| rs73167004 | A | G | 0.1811 | 0.1716 | 0.0311 | 3.59E-08 |
| rs731749 | A | G | 0.0827 | 0.276 | 0.0476 | 6.86E-09 |
| rs731839 | A | G | 0.6644 | -0.1554 | 0.0254 | 8.83E-10 |
| rs7320104 | G | A | 0.1529 | 0.3805 | 0.0342 | 1.02E-28 |
| rs73372226 | T | C | 0.0862 | -0.2586 | 0.0439 | 3.92E-09 |
| rs7341594 | A | G | 0.2197 | 0.2841 | 0.0291 | 1.55E-22 |
| rs73480560 | T | C | 0.2649 | -0.1859 | 0.0278 | 2.20E-11 |
| rs73497017 | C | G | 0.1858 | 0.1993 | 0.031 | 1.25E-10 |
| rs73530785 | G | A | 0.0776 | 0.3266 | 0.0551 | 2.99E-09 |
| rs73551577 | A | G | 0.1905 | -0.3383 | 0.0309 | 6.84E-28 |
| rs73727605 | A | G | 0.0646 | 0.2848 | 0.0511 | 2.58E-08 |
| rs73872719 | C | T | 0.0468 | 0.4615 | 0.0575 | 1.06E-15 |
| rs7395791 | A | G | 0.4415 | -0.1922 | 0.0246 | 5.26E-15 |
| rs74032414 | G | C | 0.1703 | -0.2084 | 0.0349 | 2.45E-09 |
| rs74048200 | G | A | 0.0853 | 0.3774 | 0.0453 | 8.33E-17 |
| rs741334 | G | A | 0.2899 | -0.2844 | 0.0273 | 2.41E-25 |
| rs74217009 | T | C | 0.1302 | -0.408 | 0.0397 | 8.80E-25 |
| rs74390571 | T | C | 0.1541 | -0.1851 | 0.0338 | 4.43E-08 |
| rs74516251 | A | G | 0.079 | -0.4591 | 0.0456 | 7.63E-24 |
| rs74684534 | T | C | 0.0487 | 0.3501 | 0.0599 | 4.99E-09 |
| rs74743126 | C | T | 0.0867 | -0.2619 | 0.0428 | 9.69E-10 |
| rs74900445 | C | T | 0.1196 | 0.2805 | 0.0372 | 4.58E-14 |
| rs74926649 | A | G | 0.0163 | 0.6648 | 0.0972 | 7.81E-12 |
| rs74931492 | T | C | 0.035 | 0.4606 | 0.0664 | 4.00E-12 |
| rs7500448 | G | A | 0.2517 | -0.2238 | 0.0285 | 3.81E-15 |
| rs7514579 | C | A | 0.2298 | -0.1917 | 0.0289 | 3.07E-11 |
| rs7516453 | G | C | 0.2774 | -0.2661 | 0.0271 | 8.04E-23 |
| rs7547570 | A | G | 0.3818 | 0.1897 | 0.0249 | 2.30E-14 |
| rs7562 | C | T | 0.4767 | -0.211 | 0.0243 | 4.28E-18 |
| rs75672964 | T | C | 0.043 | 0.4836 | 0.0654 | 1.43E-13 |
| rs7575637 | A | G | 0.4581 | -0.2211 | 0.0241 | 5.13E-20 |
| rs7580625 | A | G | 0.1883 | 0.2155 | 0.0307 | 2.30E-12 |
| rs7584790 | G | A | 0.4692 | -0.1371 | 0.0247 | 2.80E-08 |
| rs7603109 | C | T | 0.6654 | 0.144 | 0.0254 | 1.34E-08 |
| rs76126501 | A | G | 0.0738 | 0.2704 | 0.0464 | 5.59E-09 |
| rs7618284 | C | G | 0.3377 | -0.1732 | 0.0266 | 7.66E-11 |
| rs7632040 | G | A | 0.6031 | 0.2942 | 0.0247 | 9.71E-33 |
| rs76326501 | C | A | 0.0889 | -0.5594 | 0.0432 | 2.40E-38 |
| rs76452347 | T | C | 0.2053 | -0.2204 | 0.0309 | 9.93E-13 |
| rs7665985 | C | T | 0.6407 | -0.1411 | 0.0257 | 3.85E-08 |
| rs76719272 | T | C | 0.1286 | -0.2314 | 0.0371 | 4.40E-10 |
| rs76735299 | A | G | 0.0846 | 0.3249 | 0.0451 | 5.89E-13 |
| rs7681920 | A | C | 0.6157 | -0.1802 | 0.0249 | 4.60E-13 |
| rs7703751 | T | A | 0.2554 | -0.2518 | 0.0277 | 1.13E-19 |
| rs7710854 | G | A | 0.1171 | -0.2201 | 0.0384 | 9.74E-09 |
| rs77160800 | G | T | 0.0802 | -0.2558 | 0.045 | 1.31E-08 |
| rs7733331 | C | T | 0.5996 | 0.5493 | 0.0245 | 4.23E-111 |
| rs77347777 | T | C | 0.1182 | -0.2484 | 0.0395 | 3.35E-10 |
| rs7744498 | C | G | 0.208 | 0.1994 | 0.0297 | 1.96E-11 |
| rs775634 | G | A | 0.7713 | -0.1906 | 0.0293 | 7.76E-11 |
| rs77574378 | C | T | 0.1599 | 0.1898 | 0.0332 | 1.04E-08 |
| rs778124 | A | G | 0.3759 | 0.2513 | 0.0248 | 4.65E-24 |
| rs7784933 | A | G | 0.2791 | 0.1785 | 0.0269 | 3.51E-11 |
| rs77866798 | G | A | 0.1361 | -0.2107 | 0.0364 | 6.88E-09 |
| rs77870048 | T | C | 0.0496 | 0.7273 | 0.0662 | 4.21E-28 |
| rs77924615 | A | G | 0.1992 | -0.2961 | 0.0312 | 2.37E-21 |
| rs7796089 | C | G | 0.3387 | -0.1981 | 0.0256 | 1.10E-14 |
| rs78058190 | A | G | 0.0512 | 0.4503 | 0.0796 | 1.57E-08 |
| rs7814142 | A | G | 0.5865 | -0.329 | 0.0244 | 2.26E-41 |
| rs7822515 | A | G | 0.2662 | -0.2042 | 0.0372 | 4.03E-08 |
| rs7829347 | C | T | 0.0978 | 0.2402 | 0.0406 | 3.34E-09 |
| rs78474310 | G | A | 0.0435 | 0.4824 | 0.0593 | 4.18E-16 |
| rs785665 | A | C | 0.076 | 0.3759 | 0.0459 | 2.70E-16 |
| rs78648104 | C | T | 0.089 | 0.3218 | 0.0432 | 9.65E-14 |
| rs78670795 | A | G | 0.1002 | -0.3312 | 0.0411 | 7.98E-16 |
| rs78741089 | C | G | 0.1435 | -0.2212 | 0.0358 | 6.40E-10 |
| rs7874646 | T | C | 0.2006 | -0.1784 | 0.0299 | 2.51E-09 |
| rs79069610 | C | T | 0.0495 | 0.3206 | 0.0581 | 3.41E-08 |
| rs7912283 | A | G | 0.6473 | -0.2001 | 0.0256 | 5.88E-15 |
| rs7927515 | A | C | 0.3432 | 0.1886 | 0.026 | 3.87E-13 |
| rs7928223 | G | A | 0.262 | 0.157 | 0.0284 | 3.16E-08 |
| rs79384779 | T | C | 0.1549 | 0.3045 | 0.0344 | 8.16E-19 |
| rs79523138 | G | A | 0.1162 | 0.2713 | 0.039 | 3.30E-12 |
| rs79621605 | C | T | 0.0355 | -0.6299 | 0.0661 | 1.49E-21 |
| rs7963397 | T | C | 0.3951 | 0.1396 | 0.0246 | 1.41E-08 |
| rs7977462 | C | T | 0.7476 | -0.2039 | 0.0288 | 1.40E-12 |
| rs79909779 | A | G | 0.1382 | 0.2228 | 0.0361 | 6.93E-10 |
| rs79930761 | T | C | 0.0863 | -0.4396 | 0.0449 | 1.11E-22 |
| rs80100774 | G | T | 0.0771 | -0.3178 | 0.0512 | 5.54E-10 |
| rs8011819 | T | C | 0.4673 | -0.1629 | 0.0242 | 1.63E-11 |
| rs8013933 | C | T | 0.3046 | -0.1486 | 0.0264 | 1.80E-08 |
| rs80165581 | A | G | 0.0336 | -0.3791 | 0.0676 | 2.08E-08 |
| rs8018617 | C | A | 0.3893 | 0.2207 | 0.025 | 1.09E-18 |
| rs8032315 | A | T | 0.3009 | 0.5716 | 0.0287 | 2.90E-88 |
| rs8052276 | C | T | 0.6498 | -0.164 | 0.0261 | 3.32E-10 |
| rs8077276 | A | G | 0.6202 | -0.2313 | 0.0251 | 2.82E-20 |
| rs8102624 | A | G | 0.0793 | 0.5673 | 0.0459 | 3.76E-35 |
| rs8108717 | G | A | 0.6119 | -0.2271 | 0.0248 | 5.39E-20 |
| rs8142376 | T | C | 0.4912 | 0.1373 | 0.0239 | 9.57E-09 |
| rs817140 | C | T | 0.2758 | 0.1608 | 0.027 | 2.52E-09 |
| rs8180995 | G | A | 0.4655 | -0.1593 | 0.0242 | 4.69E-11 |
| rs839754 | T | C | 0.3827 | 0.2221 | 0.0248 | 3.27E-19 |
| rs844218 | G | A | 0.6882 | -0.1434 | 0.0261 | 4.03E-08 |
| rs848309 | C | T | 0.5705 | 0.1986 | 0.0243 | 3.42E-16 |
| rs863930 | G | T | 0.5289 | 0.1565 | 0.024 | 7.03E-11 |
| rs869396 | A | C | 0.4696 | -0.197 | 0.0245 | 8.44E-16 |
| rs869481 | A | G | 0.6617 | -0.1848 | 0.0268 | 5.23E-12 |
| rs871606 | C | T | 0.1063 | -0.3572 | 0.0401 | 5.56E-19 |
| rs872193 | T | A | 0.7148 | -0.1625 | 0.0267 | 1.19E-09 |
| rs878521 | A | G | 0.2567 | 0.1612 | 0.0285 | 1.51E-08 |
| rs880132 | C | T | 0.5733 | -0.1821 | 0.0268 | 1.04E-11 |
| rs8904 | A | G | 0.3699 | 0.2819 | 0.0249 | 1.01E-29 |
| rs9257594 | C | T | 0.7738 | -0.1892 | 0.0298 | 2.09E-10 |
| rs9286351 | G | A | 0.4214 | 0.2413 | 0.0247 | 1.59E-22 |
| rs9291932 | C | T | 0.2722 | 0.2215 | 0.0272 | 3.49E-16 |
| rs932763 | C | T | 0.6361 | 0.2035 | 0.0253 | 9.60E-16 |
| rs9337951 | A | G | 0.3387 | 0.1721 | 0.0271 | 2.26E-10 |
| rs9344215 | T | C | 0.2353 | 0.1635 | 0.0294 | 2.60E-08 |
| rs9349379 | G | A | 0.4045 | -0.2366 | 0.0247 | 9.51E-22 |
| rs9368222 | A | C | 0.2676 | 0.2276 | 0.0271 | 4.12E-17 |
| rs937276 | T | G | 0.1426 | 0.272 | 0.0345 | 2.99E-15 |
| rs937301 | G | A | 0.4428 | -0.1633 | 0.0241 | 1.28E-11 |
| rs9399953 | T | C | 0.3088 | 0.19 | 0.0329 | 7.62E-09 |
| rs9401090 | C | T | 0.2521 | -0.1717 | 0.028 | 8.50E-10 |
| rs9456648 | T | C | 0.3169 | -0.1444 | 0.026 | 2.64E-08 |
| rs949065 | A | G | 0.6997 | 0.1571 | 0.027 | 6.23E-09 |
| rs950196 | A | T | 0.1424 | 0.2811 | 0.0351 | 1.25E-15 |
| rs9506725 | C | T | 0.3659 | -0.2303 | 0.0248 | 1.60E-20 |
| rs9507866 | A | C | 0.363 | -0.1732 | 0.0254 | 9.26E-12 |
| rs9508495 | T | C | 0.7555 | -0.3111 | 0.0281 | 1.60E-28 |
| rs9515270 | T | C | 0.1779 | 0.1815 | 0.0318 | 1.19E-08 |
| rs9527686 | G | A | 0.3672 | -0.1476 | 0.0253 | 5.78E-09 |
| rs9530914 | T | G | 0.3962 | 0.148 | 0.0253 | 4.95E-09 |
| rs9532243 | C | A | 0.5185 | -0.2679 | 0.024 | 5.58E-29 |
| rs9532959 | A | G | 0.0798 | 0.2705 | 0.046 | 4.14E-09 |
| rs954767 | C | A | 0.259 | 0.1915 | 0.0274 | 2.97E-12 |
| rs955683 | C | T | 0.5046 | 0.1793 | 0.0257 | 2.91E-12 |
| rs9596839 | A | G | 0.2912 | -0.1553 | 0.0267 | 5.87E-09 |
| rs9630625 | T | C | 0.1177 | 0.2812 | 0.0387 | 3.77E-13 |
| rs9634314 | A | G | 0.0802 | -0.346 | 0.0451 | 1.78E-14 |
| rs9636439 | T | C | 0.7644 | 0.2023 | 0.0285 | 1.37E-12 |
| rs9667596 | C | T | 0.1401 | -0.4367 | 0.0367 | 1.30E-32 |
| rs9675039 | A | G | 0.3673 | 0.1459 | 0.0255 | 1.07E-08 |
| rs9729149 | T | C | 0.4223 | -0.2281 | 0.0248 | 3.49E-20 |
| rs9766666 | T | G | 0.2712 | -0.1622 | 0.0273 | 2.94E-09 |
| rs977184 | C | T | 0.3688 | 0.1711 | 0.0253 | 1.40E-11 |
| rs9809556 | A | G | 0.3925 | 0.2923 | 0.0247 | 2.21E-32 |
| rs9811721 | C | T | 0.6898 | 0.1437 | 0.0263 | 4.54E-08 |
| rs981883 | G | A | 0.6121 | 0.1668 | 0.0247 | 1.55E-11 |
| rs9833313 | T | A | 0.757 | 0.1579 | 0.0285 | 3.06E-08 |
| rs9841978 | A | G | 0.3259 | 0.2072 | 0.0261 | 1.95E-15 |
| rs9842387 | G | A | 0.4633 | 0.1329 | 0.0242 | 4.15E-08 |
| rs9849022 | T | G | 0.5135 | 0.1414 | 0.024 | 3.90E-09 |
| rs9877020 | T | C | 0.1609 | 0.184 | 0.033 | 2.57E-08 |
| rs988398 | T | C | 0.4424 | 0.3037 | 0.0242 | 3.28E-36 |
| rs9885577 | T | C | 0.3723 | 0.2069 | 0.0257 | 9.31E-16 |
| rs9885632 | T | C | 0.7317 | 0.2009 | 0.0274 | 2.33E-13 |
| rs9886665 | C | T | 0.7259 | -0.1629 | 0.0275 | 3.36E-09 |
| rs9886857 | A | G | 0.1512 | -0.1871 | 0.0338 | 3.13E-08 |
| rs9899540 | T | A | 0.5969 | -0.1699 | 0.0253 | 1.82E-11 |
| rs9926191 | G | A | 0.0751 | 0.3021 | 0.0494 | 9.77E-10 |
| rs9934511 | A | T | 0.0667 | -0.293 | 0.0489 | 2.09E-09 |
| rs9937801 | C | T | 0.4295 | -0.1755 | 0.0241 | 3.30E-13 |

**Supplementary Table 2. Genetic variants used to proxy effects of diastolic blood pressure.**

Table is provided at end of document. EA: effect allele; EAF: effect allele frequency; OA: other allele; SE: standard error; SNP: single nucleotide polymorphism.

| **SNP** | **EA** | **OA** | **EAF** | **Beta** | **SE** | **P** |
| --- | --- | --- | --- | --- | --- | --- |
| rs10004996 | C | T | 0.5346 | -0.1141 | 0.0151 | 3.90E-14 |
| rs1004291 | A | G | 0.4248 | 0.1282 | 0.0151 | 2.37E-17 |
| rs10048404 | T | C | 0.3668 | -0.0973 | 0.0164 | 2.79E-09 |
| rs1006100 | T | C | 0.7978 | 0.1628 | 0.019 | 9.59E-18 |
| rs10062049 | T | C | 0.136 | 0.2115 | 0.0221 | 9.62E-22 |
| rs10066799 | T | G | 0.28 | 0.1167 | 0.0167 | 3.07E-12 |
| rs10069690 | T | C | 0.2583 | 0.1475 | 0.0178 | 1.29E-16 |
| rs10087280 | G | A | 0.1712 | -0.1273 | 0.02 | 1.86E-10 |
| rs10112148 | C | T | 0.5408 | -0.1175 | 0.0153 | 1.34E-14 |
| rs10160846 | T | C | 0.1584 | 0.1498 | 0.021 | 9.13E-13 |
| rs10167465 | A | G | 0.6279 | -0.1807 | 0.0157 | 1.33E-30 |
| rs10170954 | C | A | 0.6504 | 0.0963 | 0.0156 | 7.63E-10 |
| rs10192654 | T | A | 0.2767 | 0.1231 | 0.0169 | 2.99E-13 |
| rs10201722 | T | G | 0.5134 | 0.1338 | 0.0151 | 6.95E-19 |
| rs10233127 | A | T | 0.109 | 0.1569 | 0.0251 | 3.88E-10 |
| rs10251492 | A | G | 0.6377 | 0.1167 | 0.0159 | 1.95E-13 |
| rs1035272 | T | G | 0.8997 | 0.1587 | 0.0254 | 4.08E-10 |
| rs1035514 | A | G | 0.8756 | 0.1569 | 0.0227 | 4.40E-12 |
| rs10409243 | T | C | 0.5883 | -0.1125 | 0.0157 | 8.06E-13 |
| rs10437954 | A | G | 0.9045 | -0.1752 | 0.0263 | 2.64E-11 |
| rs1044145 | C | T | 0.5461 | -0.1061 | 0.0152 | 2.57E-12 |
| rs1044608 | G | C | 0.076 | 0.1959 | 0.0297 | 4.49E-11 |
| rs1044822 | T | C | 0.148 | -0.1292 | 0.0212 | 1.13E-09 |
| rs10468291 | A | C | 0.5695 | -0.0946 | 0.0153 | 6.42E-10 |
| rs1047891 | A | C | 0.3183 | -0.1124 | 0.0162 | 3.98E-12 |
| rs10493408 | A | C | 0.134 | 0.1503 | 0.022 | 8.07E-12 |
| rs10495928 | G | A | 0.3348 | -0.1185 | 0.0157 | 5.19E-14 |
| rs1049724 | T | C | 0.0421 | 0.2457 | 0.0394 | 4.32E-10 |
| rs1052486 | G | A | 0.4795 | 0.2232 | 0.0153 | 4.91E-48 |
| rs1060206 | C | T | 0.7237 | 0.1153 | 0.017 | 1.09E-11 |
| rs10650 | G | C | 0.2529 | -0.1407 | 0.0173 | 3.72E-16 |
| rs10739693 | A | G | 0.3027 | 0.0934 | 0.0166 | 1.98E-08 |
| rs10747570 | G | A | 0.6218 | -0.2489 | 0.0154 | 7.58E-59 |
| rs10759344 | T | C | 0.9145 | 0.1703 | 0.0273 | 4.64E-10 |
| rs10759697 | A | G | 0.4917 | 0.1181 | 0.015 | 2.85E-15 |
| rs10761530 | C | T | 0.5021 | -0.0964 | 0.015 | 1.14E-10 |
| rs10766309 | G | A | 0.7973 | -0.2869 | 0.0186 | 1.49E-53 |
| rs10776752 | T | G | 0.0785 | 0.4241 | 0.0286 | 9.00E-50 |
| rs10779426 | T | A | 0.5835 | 0.1053 | 0.0152 | 4.08E-12 |
| rs10804330 | C | T | 0.433 | -0.1336 | 0.0153 | 2.69E-18 |
| rs10812133 | G | A | 0.1998 | -0.1178 | 0.0188 | 3.43E-10 |
| rs10819246 | T | G | 0.0991 | 0.1662 | 0.0254 | 5.91E-11 |
| rs10832300 | G | A | 0.4122 | 0.1031 | 0.0152 | 1.30E-11 |
| rs10835186 | A | G | 0.7301 | -0.1271 | 0.017 | 7.88E-14 |
| rs10838702 | T | G | 0.3875 | 0.2156 | 0.0154 | 1.98E-44 |
| rs10840378 | T | C | 0.1889 | 0.2559 | 0.0191 | 6.99E-41 |
| rs10842709 | G | T | 0.3315 | -0.1332 | 0.0159 | 6.64E-17 |
| rs10846681 | A | G | 0.1705 | 0.1361 | 0.0219 | 5.01E-10 |
| rs10850526 | A | G | 0.3009 | -0.1614 | 0.0164 | 7.59E-23 |
| rs10864859 | T | G | 0.9149 | 0.1804 | 0.0281 | 1.41E-10 |
| rs10872410 | T | C | 0.049 | -0.2176 | 0.0352 | 6.53E-10 |
| rs10889711 | C | T | 0.6321 | -0.0905 | 0.0156 | 6.57E-09 |
| rs10906391 | T | C | 0.3187 | 0.1258 | 0.0163 | 1.15E-14 |
| rs10941043 | G | T | 0.2889 | 0.0961 | 0.0165 | 5.32E-09 |
| rs10988229 | C | T | 0.2107 | -0.1058 | 0.0187 | 1.57E-08 |
| rs11021221 | A | T | 0.165 | -0.184 | 0.0202 | 7.39E-20 |
| rs11026586 | A | G | 0.0701 | 0.2633 | 0.0301 | 2.04E-18 |
| rs11029977 | C | T | 0.6117 | -0.1216 | 0.0154 | 3.37E-15 |
| rs11030112 | A | G | 0.314 | -0.1547 | 0.0161 | 8.40E-22 |
| rs11059094 | T | C | 0.4765 | -0.1343 | 0.015 | 4.59E-19 |
| rs11065078 | T | C | 0.2091 | 0.1398 | 0.0188 | 9.16E-14 |
| rs11065129 | G | A | 0.4221 | -0.1195 | 0.0154 | 8.00E-15 |
| rs11070245 | G | T | 0.5315 | 0.1264 | 0.0151 | 5.41E-17 |
| rs11077961 | G | A | 0.3699 | -0.0895 | 0.016 | 2.09E-08 |
| rs11082866 | T | A | 0.2978 | 0.1173 | 0.0167 | 1.89E-12 |
| rs11083560 | G | C | 0.2733 | 0.1354 | 0.0171 | 2.34E-15 |
| rs11084997 | C | G | 0.3751 | -0.0908 | 0.0162 | 1.92E-08 |
| rs11085015 | G | T | 0.8036 | 0.1503 | 0.0244 | 7.31E-10 |
| rs11112548 | T | A | 0.044 | -0.2621 | 0.0388 | 1.46E-11 |
| rs11124186 | C | G | 0.212 | 0.1107 | 0.0184 | 1.84E-09 |
| rs11125883 | C | A | 0.3725 | -0.1047 | 0.0155 | 1.41E-11 |
| rs11141731 | T | C | 0.2246 | -0.1062 | 0.0181 | 4.54E-09 |
| rs1114347 | G | A | 0.4804 | 0.1681 | 0.0151 | 9.15E-29 |
| rs11145807 | G | A | 0.5909 | -0.1255 | 0.0159 | 2.76E-15 |
| rs11153730 | C | T | 0.4893 | -0.1346 | 0.0149 | 1.71E-19 |
| rs11155745 | A | G | 0.3333 | 0.0957 | 0.016 | 2.29E-09 |
| rs111777102 | T | C | 0.0661 | 0.186 | 0.0307 | 1.44E-09 |
| rs111821611 | A | G | 0.5086 | -0.108 | 0.018 | 2.07E-09 |
| rs11187838 | A | G | 0.4348 | -0.2507 | 0.015 | 2.70E-62 |
| rs11191614 | T | C | 0.1396 | 0.1909 | 0.0218 | 1.76E-18 |
| rs11191801 | C | A | 0.2955 | 0.1053 | 0.0165 | 1.59E-10 |
| rs11196549 | A | G | 0.0439 | 0.3677 | 0.0381 | 5.02E-22 |
| rs11224683 | T | C | 0.1729 | -0.1132 | 0.0198 | 1.05E-08 |
| rs112252760 | A | G | 0.3039 | 0.0919 | 0.0165 | 2.32E-08 |
| rs11228953 | T | G | 0.1289 | -0.2065 | 0.0245 | 3.78E-17 |
| rs11235770 | T | C | 0.1193 | -0.1364 | 0.0232 | 4.11E-09 |
| rs11241305 | A | C | 0.4894 | 0.1385 | 0.015 | 2.39E-20 |
| rs11244061 | T | C | 0.1139 | -0.2273 | 0.0238 | 1.14E-21 |
| rs11246486 | T | C | 0.1312 | -0.2304 | 0.026 | 8.55E-19 |
| rs11252324 | T | G | 0.0777 | -0.2067 | 0.0283 | 2.57E-13 |
| rs112873509 | C | T | 0.1085 | 0.1557 | 0.0245 | 2.15E-10 |
| rs112993339 | T | C | 0.0456 | -0.2591 | 0.0377 | 6.58E-12 |
| rs113134141 | G | A | 0.1019 | 0.1526 | 0.0249 | 8.74E-10 |
| rs1133400 | G | A | 0.214 | 0.1131 | 0.0185 | 9.63E-10 |
| rs113783450 | A | G | 0.3133 | -0.116 | 0.0165 | 2.23E-12 |
| rs1138293 | T | C | 0.196 | -0.1373 | 0.019 | 4.52E-13 |
| rs114503346 | T | C | 0.0447 | -0.2488 | 0.0373 | 2.45E-11 |
| rs114714860 | C | G | 0.1706 | 0.3144 | 0.0202 | 1.15E-54 |
| rs11543651 | C | T | 0.3233 | 0.0965 | 0.0164 | 3.96E-09 |
| rs11556924 | T | C | 0.3818 | -0.1692 | 0.0156 | 1.79E-27 |
| rs11604175 | T | C | 0.2558 | 0.1037 | 0.0173 | 1.99E-09 |
| rs11605229 | T | C | 0.1075 | -0.2524 | 0.0245 | 8.39E-25 |
| rs11608149 | A | G | 0.2665 | 0.1115 | 0.0171 | 6.79E-11 |
| rs11610535 | G | A | 0.0985 | 0.1392 | 0.0253 | 3.77E-08 |
| rs11618950 | A | G | 0.1722 | -0.1157 | 0.02 | 7.20E-09 |
| rs11624528 | A | G | 0.4089 | 0.0864 | 0.0154 | 2.25E-08 |
| rs11628933 | C | G | 0.2334 | -0.1359 | 0.0178 | 2.18E-14 |
| rs116294778 | A | G | 0.0342 | -0.2489 | 0.0421 | 3.48E-09 |
| rs11634028 | A | T | 0.2104 | 0.1203 | 0.0192 | 3.64E-10 |
| rs116479312 | G | T | 0.0199 | -0.4547 | 0.0672 | 1.32E-11 |
| rs11665020 | C | G | 0.32 | -0.1334 | 0.0163 | 2.39E-16 |
| rs116760613 | A | G | 0.0185 | -0.4137 | 0.0569 | 3.67E-13 |
| rs1168580 | T | C | 0.4816 | 0.0977 | 0.015 | 7.59E-11 |
| rs116902041 | T | C | 0.1022 | 0.145 | 0.0255 | 1.24E-08 |
| rs116909641 | T | C | 0.0468 | -0.3597 | 0.0489 | 1.86E-13 |
| rs11692619 | T | C | 0.3559 | -0.1102 | 0.0164 | 1.93E-11 |
| rs11694714 | T | C | 0.3298 | 0.0865 | 0.0158 | 4.24E-08 |
| rs11696852 | T | C | 0.232 | 0.1175 | 0.0179 | 5.72E-11 |
| rs117311861 | T | C | 0.0225 | 0.3113 | 0.0525 | 3.09E-09 |
| rs11738827 | T | C | 0.3682 | -0.1242 | 0.0167 | 1.21E-13 |
| rs11757455 | A | G | 0.0662 | -0.1734 | 0.0303 | 1.03E-08 |
| rs117604516 | A | G | 0.1199 | 0.2047 | 0.0239 | 9.99E-18 |
| rs1177764 | G | C | 0.5954 | 0.2831 | 0.0152 | 5.24E-77 |
| rs117777118 | A | G | 0.0399 | -0.3583 | 0.0489 | 2.40E-13 |
| rs11778153 | C | T | 0.3557 | -0.1111 | 0.0157 | 1.69E-12 |
| rs11781001 | A | G | 0.1556 | 0.1261 | 0.0208 | 1.33E-09 |
| rs117828113 | C | T | 0.0662 | -0.1929 | 0.0308 | 3.71E-10 |
| rs1178979 | C | T | 0.1935 | -0.1425 | 0.0191 | 8.17E-14 |
| rs117913411 | A | T | 0.0373 | 0.378 | 0.0421 | 2.95E-19 |
| rs11853359 | A | G | 0.333 | -0.1588 | 0.0159 | 1.43E-23 |
| rs11856577 | C | T | 0.4661 | 0.1623 | 0.0152 | 1.49E-26 |
| rs11857515 | A | T | 0.2041 | 0.1355 | 0.0195 | 3.78E-12 |
| rs11859505 | G | A | 0.5751 | 0.0897 | 0.0158 | 1.29E-08 |
| rs11882467 | T | G | 0.293 | 0.1017 | 0.0166 | 9.98E-10 |
| rs11899825 | T | G | 0.5668 | -0.0968 | 0.0153 | 2.25E-10 |
| rs11899888 | G | A | 0.1527 | 0.1272 | 0.0211 | 1.77E-09 |
| rs11956654 | G | A | 0.2595 | -0.1221 | 0.017 | 7.40E-13 |
| rs11960210 | C | T | 0.3793 | -0.2148 | 0.0156 | 4.39E-43 |
| rs11998678 | T | C | 0.4721 | 0.1387 | 0.0152 | 9.12E-20 |
| rs12052761 | A | G | 0.3954 | -0.1001 | 0.0153 | 6.82E-11 |
| rs12078697 | C | G | 0.2116 | -0.1058 | 0.0184 | 9.03E-09 |
| rs12097553 | C | T | 0.0765 | 0.1974 | 0.0287 | 6.02E-12 |
| rs12114418 | G | A | 0.2344 | 0.1291 | 0.0179 | 5.68E-13 |
| rs12119765 | C | T | 0.5218 | -0.0945 | 0.015 | 3.20E-10 |
| rs1212061 | C | G | 0.7323 | 0.1136 | 0.0171 | 2.75E-11 |
| rs12149258 | G | A | 0.1587 | -0.1221 | 0.0207 | 3.47E-09 |
| rs1215469 | C | A | 0.7713 | 0.1163 | 0.0183 | 2.01E-10 |
| rs1220128 | C | G | 0.8512 | 0.1762 | 0.0211 | 8.01E-17 |
| rs12208884 | A | G | 0.1889 | 0.1316 | 0.0191 | 5.98E-12 |
| rs12255289 | T | C | 0.2261 | 0.1082 | 0.0178 | 1.28E-09 |
| rs12258967 | G | C | 0.2969 | -0.3226 | 0.0168 | 1.82E-82 |
| rs12402865 | C | T | 0.6245 | -0.0886 | 0.016 | 2.99E-08 |
| rs12405515 | T | G | 0.572 | -0.1451 | 0.0151 | 7.36E-22 |
| rs12413835 | G | T | 0.0529 | 0.2334 | 0.0358 | 6.95E-11 |
| rs12414028 | A | T | 0.0873 | -0.4506 | 0.0277 | 2.63E-59 |
| rs12433714 | G | A | 0.4332 | -0.0847 | 0.0152 | 2.54E-08 |
| rs12437164 | C | T | 0.3462 | -0.1024 | 0.016 | 1.50E-10 |
| rs1243876 | T | C | 0.7017 | -0.1057 | 0.0165 | 1.67E-10 |
| rs12446456 | T | C | 0.4288 | -0.1703 | 0.0151 | 2.40E-29 |
| rs12463045 | C | T | 0.8305 | -0.1579 | 0.0209 | 4.23E-14 |
| rs12482569 | A | G | 0.1727 | -0.1257 | 0.0199 | 2.66E-10 |
| rs12509595 | C | T | 0.2918 | 0.4536 | 0.0165 | 6.56E-166 |
| rs12515541 | T | G | 0.61 | 0.1073 | 0.0154 | 2.73E-12 |
| rs12520730 | C | T | 0.6743 | 0.0949 | 0.0161 | 4.22E-09 |
| rs12523151 | C | T | 0.5734 | 0.0887 | 0.0154 | 8.86E-09 |
| rs12535680 | G | A | 0.3378 | 0.1177 | 0.0164 | 7.93E-13 |
| rs12536606 | T | C | 0.2686 | -0.0987 | 0.0171 | 7.36E-09 |
| rs12542733 | G | T | 0.4724 | 0.143 | 0.0151 | 2.67E-21 |
| rs12583637 | G | C | 0.1319 | 0.1767 | 0.0224 | 3.40E-15 |
| rs12593086 | C | G | 0.0666 | -0.1694 | 0.0308 | 4.00E-08 |
| rs12601936 | G | A | 0.6079 | 0.1351 | 0.0155 | 3.47E-18 |
| rs12609484 | T | G | 0.3121 | -0.137 | 0.0164 | 5.98E-17 |
| rs12623637 | G | A | 0.4444 | 0.1045 | 0.0152 | 6.23E-12 |
| rs12627514 | G | C | 0.2881 | 0.1874 | 0.0171 | 7.91E-28 |
| rs1263671 | C | T | 0.1634 | 0.123 | 0.0207 | 3.05E-09 |
| rs1265842 | C | T | 0.5159 | -0.0996 | 0.0152 | 4.96E-11 |
| rs12676005 | G | C | 0.197 | 0.1277 | 0.0188 | 1.10E-11 |
| rs1268539 | A | C | 0.4225 | -0.1115 | 0.0153 | 2.70E-13 |
| rs12693302 | A | G | 0.6512 | -0.207 | 0.0156 | 4.89E-40 |
| rs12712893 | G | A | 0.6322 | -0.1044 | 0.0156 | 2.03E-11 |
| rs12730750 | A | G | 0.3397 | 0.1099 | 0.0164 | 1.92E-11 |
| rs1275988 | T | C | 0.6112 | -0.2867 | 0.0153 | 9.22E-79 |
| rs12790943 | T | C | 0.4205 | -0.0878 | 0.0152 | 7.51E-09 |
| rs12823424 | G | A | 0.2821 | 0.1171 | 0.0166 | 1.68E-12 |
| rs12883344 | A | C | 0.3984 | 0.0833 | 0.0153 | 4.94E-08 |
| rs12888994 | C | A | 0.5657 | -0.0954 | 0.0152 | 3.68E-10 |
| rs12905199 | G | A | 0.6678 | -0.3812 | 0.018 | 1.76E-99 |
| rs12906962 | C | T | 0.325 | 0.2057 | 0.0163 | 1.21E-36 |
| rs12912791 | C | T | 0.1722 | -0.134 | 0.0198 | 1.27E-11 |
| rs12919839 | T | C | 0.2856 | -0.0994 | 0.0166 | 2.15E-09 |
| rs12930988 | A | T | 0.6432 | 0.1466 | 0.0158 | 1.94E-20 |
| rs12941755 | G | C | 0.2355 | 0.1026 | 0.0178 | 8.21E-09 |
| rs12978472 | G | C | 0.1238 | -0.4373 | 0.0241 | 1.35E-73 |
| rs12983238 | G | A | 0.6946 | 0.1152 | 0.0176 | 6.07E-11 |
| rs13107325 | T | C | 0.0759 | -0.6118 | 0.0288 | 4.68E-100 |
| rs13150093 | A | G | 0.4793 | -0.1362 | 0.0152 | 3.30E-19 |
| rs13156484 | A | G | 0.4748 | 0.1826 | 0.0152 | 2.06E-33 |
| rs13179413 | T | C | 0.281 | 0.1319 | 0.0173 | 2.45E-14 |
| rs13195550 | T | C | 0.3186 | 0.1322 | 0.0163 | 5.68E-16 |
| rs13215166 | G | A | 0.4412 | 0.2781 | 0.0151 | 3.39E-76 |
| rs13217066 | T | C | 0.4049 | 0.0898 | 0.0153 | 4.41E-09 |
| rs1322639 | A | G | 0.7748 | -0.1576 | 0.0181 | 3.22E-18 |
| rs13228123 | T | G | 0.2614 | 0.0976 | 0.0171 | 1.05E-08 |
| rs13230519 | A | G | 0.1613 | 0.1478 | 0.0255 | 6.81E-09 |
| rs13270541 | A | G | 0.2192 | 0.1157 | 0.0182 | 2.18E-10 |
| rs1327235 | G | A | 0.47 | 0.2795 | 0.0149 | 4.23E-78 |
| rs1332813 | C | T | 0.6492 | -0.1125 | 0.0159 | 1.41E-12 |
| rs13358657 | G | A | 0.1321 | 0.1967 | 0.0222 | 6.80E-19 |
| rs13401387 | T | C | 0.4525 | -0.0979 | 0.0152 | 1.31E-10 |
| rs13420463 | G | A | 0.2305 | -0.1391 | 0.0178 | 5.02E-15 |
| rs1347345 | A | G | 0.6165 | -0.0948 | 0.0155 | 1.03E-09 |
| rs1348792 | G | A | 0.3813 | -0.1207 | 0.0175 | 4.67E-12 |
| rs1373780 | C | G | 0.1846 | 0.1096 | 0.0193 | 1.33E-08 |
| rs140450269 | A | G | 0.0799 | 0.1597 | 0.0279 | 1.01E-08 |
| rs1407256 | G | A | 0.1553 | 0.1186 | 0.0213 | 2.67E-08 |
| rs141212865 | C | A | 0.1981 | -0.1392 | 0.0191 | 3.14E-13 |
| rs142449193 | T | C | 0.0458 | -0.2222 | 0.0374 | 2.84E-09 |
| rs1425486 | T | C | 0.3232 | -0.1227 | 0.0161 | 2.45E-14 |
| rs1436138 | G | A | 0.3659 | -0.1611 | 0.0157 | 1.03E-24 |
| rs1446468 | C | T | 0.542 | 0.2376 | 0.0151 | 1.52E-55 |
| rs145301271 | T | C | 0.026 | 0.2915 | 0.0477 | 9.95E-10 |
| rs145316067 | A | G | 0.035 | -0.2395 | 0.0438 | 4.44E-08 |
| rs145874315 | T | C | 0.0577 | -0.2058 | 0.0338 | 1.15E-09 |
| rs146336654 | A | G | 0.0351 | 0.2413 | 0.0427 | 1.58E-08 |
| rs146385050 | A | C | 0.1751 | -0.1299 | 0.0222 | 5.08E-09 |
| rs146827176 | T | C | 0.048 | -0.2047 | 0.0368 | 2.75E-08 |
| rs1469789 | C | A | 0.2593 | 0.1149 | 0.017 | 1.59E-11 |
| rs147468572 | A | G | 0.1242 | -0.1247 | 0.0227 | 4.13E-08 |
| rs148221695 | C | T | 0.0441 | 0.2962 | 0.0378 | 5.05E-15 |
| rs148360817 | C | A | 0.0239 | 0.3048 | 0.0538 | 1.43E-08 |
| rs148590717 | T | C | 0.0738 | 0.1627 | 0.0295 | 3.59E-08 |
| rs149866169 | A | T | 0.1139 | -0.2445 | 0.0238 | 9.99E-25 |
| rs150816167 | C | T | 0.0455 | 0.2629 | 0.0385 | 9.03E-12 |
| rs1527351 | C | T | 0.338 | -0.1624 | 0.0159 | 1.25E-24 |
| rs1534338 | A | G | 0.6056 | -0.1063 | 0.0154 | 5.61E-12 |
| rs1543310 | C | T | 0.541 | 0.1181 | 0.0156 | 3.47E-14 |
| rs1546722 | G | A | 0.5177 | -0.0866 | 0.015 | 7.46E-09 |
| rs1558259 | G | A | 0.784 | -0.1129 | 0.0183 | 6.20E-10 |
| rs1569844 | A | G | 0.4486 | 0.1126 | 0.015 | 5.28E-14 |
| rs1571737 | C | T | 0.1751 | -0.1241 | 0.0199 | 5.01E-10 |
| rs160838 | A | G | 0.5854 | 0.1906 | 0.0198 | 7.16E-22 |
| rs1623474 | T | C | 0.3314 | 0.1999 | 0.0159 | 4.74E-36 |
| rs1665672 | T | G | 0.6989 | -0.0904 | 0.0164 | 3.56E-08 |
| rs1669907 | G | T | 0.6968 | -0.1112 | 0.0164 | 1.36E-11 |
| rs1675383 | A | C | 0.4427 | 0.1416 | 0.0151 | 5.58E-21 |
| rs16848690 | T | C | 0.1036 | 0.1533 | 0.0249 | 7.48E-10 |
| rs16853198 | G | A | 0.0765 | -0.3006 | 0.0287 | 1.04E-25 |
| rs1687295 | C | T | 0.7288 | -0.1841 | 0.0168 | 7.19E-28 |
| rs16875501 | T | C | 0.0672 | 0.1672 | 0.0301 | 2.78E-08 |
| rs1694068 | A | T | 0.6157 | 0.1299 | 0.0154 | 3.66E-17 |
| rs17035646 | A | G | 0.3417 | 0.2596 | 0.0159 | 1.14E-59 |
| rs1706003 | T | G | 0.4645 | 0.1317 | 0.0156 | 2.97E-17 |
| rs17080093 | T | C | 0.0707 | -0.4293 | 0.0293 | 9.45E-49 |
| rs17162311 | G | A | 0.0798 | 0.1983 | 0.0277 | 7.77E-13 |
| rs17184754 | T | C | 0.0311 | -0.2506 | 0.0459 | 4.78E-08 |
| rs17224476 | A | G | 0.111 | 0.1459 | 0.0239 | 9.67E-10 |
| rs1727311 | G | T | 0.8023 | 0.1787 | 0.0191 | 8.44E-21 |
| rs172906 | C | A | 0.5574 | 0.0951 | 0.0164 | 7.13E-09 |
| rs1732235 | C | T | 0.4987 | -0.0867 | 0.015 | 8.18E-09 |
| rs17362588 | A | G | 0.0886 | 0.3235 | 0.0263 | 6.94E-35 |
| rs173692 | G | A | 0.5777 | 0.0834 | 0.0153 | 4.68E-08 |
| rs17396055 | A | G | 0.3299 | -0.1049 | 0.0161 | 7.07E-11 |
| rs17574630 | A | G | 0.1967 | -0.1245 | 0.0191 | 6.27E-11 |
| rs17622656 | A | G | 0.3766 | -0.1397 | 0.0156 | 4.04E-19 |
| rs1764975 | A | T | 0.7983 | 0.1406 | 0.0189 | 1.09E-13 |
| rs17657522 | A | G | 0.5244 | -0.0882 | 0.0153 | 7.70E-09 |
| rs17678552 | C | T | 0.3444 | 0.1566 | 0.0158 | 2.91E-23 |
| rs17696741 | A | G | 0.1225 | 0.1323 | 0.0234 | 1.50E-08 |
| rs17732513 | T | C | 0.3463 | 0.1103 | 0.0161 | 7.71E-12 |
| rs17742342 | C | A | 0.1999 | -0.1442 | 0.0187 | 1.21E-14 |
| rs1814826 | G | T | 0.3963 | 0.0882 | 0.0153 | 8.33E-09 |
| rs1821295 | T | C | 0.6991 | -0.142 | 0.0164 | 4.83E-18 |
| rs1867624 | T | C | 0.6145 | 0.1022 | 0.0156 | 5.03E-11 |
| rs1870839 | G | A | 0.2657 | -0.1133 | 0.0171 | 3.71E-11 |
| rs1871190 | T | G | 0.333 | 0.0911 | 0.0162 | 2.02E-08 |
| rs1871813 | A | T | 0.3962 | 0.0894 | 0.0161 | 2.99E-08 |
| rs1876489 | A | G | 0.7153 | 0.1133 | 0.0166 | 1.00E-11 |
| rs1878825 | G | C | 0.3582 | 0.101 | 0.0158 | 1.83E-10 |
| rs1879056 | T | C | 0.7917 | -0.2101 | 0.0192 | 7.72E-28 |
| rs1882961 | T | C | 0.3074 | 0.1116 | 0.0164 | 1.12E-11 |
| rs1885018 | C | A | 0.1654 | -0.1221 | 0.0202 | 1.63E-09 |
| rs1906672 | A | G | 0.2325 | 0.1191 | 0.0178 | 2.06E-11 |
| rs1907400 | T | C | 0.4845 | 0.1325 | 0.0149 | 6.95E-19 |
| rs1950500 | C | T | 0.709 | -0.1278 | 0.0164 | 7.25E-15 |
| rs195486 | C | T | 0.3781 | -0.1214 | 0.0155 | 4.44E-15 |
| rs1984195 | A | G | 0.4881 | 0.1717 | 0.0151 | 6.53E-30 |
| rs198851 | G | T | 0.8507 | -0.3517 | 0.021 | 9.36E-63 |
| rs2029134 | G | T | 0.6488 | 0.0955 | 0.0156 | 9.71E-10 |
| rs2048976 | C | G | 0.2867 | -0.0984 | 0.0168 | 4.60E-09 |
| rs2070527 | C | A | 0.7508 | 0.1334 | 0.0175 | 2.30E-14 |
| rs2089760 | T | C | 0.5235 | -0.101 | 0.015 | 1.92E-11 |
| rs2095365 | T | C | 0.3172 | 0.0896 | 0.0161 | 2.84E-08 |
| rs2098839 | C | T | 0.6669 | -0.165 | 0.0159 | 2.90E-25 |
| rs2125578 | T | C | 0.5383 | -0.0833 | 0.015 | 2.70E-08 |
| rs214080 | G | A | 0.5829 | 0.0895 | 0.0152 | 3.96E-09 |
| rs2144249 | C | T | 0.15 | -0.128 | 0.0211 | 1.20E-09 |
| rs2161967 | G | T | 0.5714 | -0.157 | 0.0153 | 8.47E-25 |
| rs2171690 | C | T | 0.4622 | -0.1074 | 0.0151 | 1.19E-12 |
| rs2173030 | C | G | 0.8887 | 0.1931 | 0.0241 | 1.11E-15 |
| rs2179129 | G | A | 0.4137 | -0.129 | 0.0152 | 2.54E-17 |
| rs220249 | A | G | 0.4487 | 0.0878 | 0.0151 | 6.50E-09 |
| rs2208589 | G | A | 0.7748 | 0.1778 | 0.0187 | 1.99E-21 |
| rs223102 | T | C | 0.526 | -0.2443 | 0.0149 | 2.46E-60 |
| rs2236295 | T | G | 0.3963 | -0.187 | 0.0154 | 8.25E-34 |
| rs2239268 | A | G | 0.6993 | 0.1242 | 0.0163 | 3.03E-14 |
| rs2240075 | A | G | 0.7254 | -0.1294 | 0.0171 | 4.19E-14 |
| rs2240736 | T | C | 0.7308 | 0.1812 | 0.017 | 1.75E-26 |
| rs2244386 | A | C | 0.4807 | 0.0915 | 0.015 | 9.89E-10 |
| rs2256187 | A | G | 0.1584 | -0.1198 | 0.0205 | 5.48E-09 |
| rs2269579 | G | A | 0.1018 | 0.2029 | 0.0249 | 3.32E-16 |
| rs2273171 | C | T | 0.4736 | 0.0854 | 0.0149 | 1.10E-08 |
| rs227426 | T | G | 0.5607 | 0.0877 | 0.0152 | 8.18E-09 |
| rs2276626 | T | C | 0.6351 | 0.1142 | 0.0157 | 3.34E-13 |
| rs2282527 | A | C | 0.3414 | 0.1019 | 0.0168 | 1.37E-09 |
| rs2289123 | T | G | 0.7726 | -0.1115 | 0.0187 | 2.68E-09 |
| rs2293476 | C | G | 0.2272 | 0.116 | 0.0179 | 1.01E-10 |
| rs2298359 | C | T | 0.0618 | -0.2326 | 0.0359 | 9.05E-11 |
| rs229872 | T | G | 0.7343 | -0.0936 | 0.0171 | 4.76E-08 |
| rs2298755 | G | C | 0.4134 | 0.0855 | 0.0152 | 1.67E-08 |
| rs2301597 | C | T | 0.5696 | -0.1466 | 0.0151 | 3.75E-22 |
| rs2306363 | T | G | 0.2025 | -0.2349 | 0.0187 | 3.79E-36 |
| rs2306623 | C | T | 0.6697 | 0.0929 | 0.0159 | 5.22E-09 |
| rs2307111 | C | T | 0.3972 | 0.1723 | 0.0154 | 3.41E-29 |
| rs2314086 | A | G | 0.3506 | 0.0927 | 0.0158 | 4.25E-09 |
| rs2320590 | T | C | 0.5499 | 0.0851 | 0.015 | 1.38E-08 |
| rs2355702 | C | T | 0.1305 | 0.1261 | 0.0223 | 1.59E-08 |
| rs2379120 | T | A | 0.711 | -0.0999 | 0.0172 | 6.68E-09 |
| rs2427529 | A | T | 0.2382 | -0.1301 | 0.0176 | 1.60E-13 |
| rs2442618 | C | T | 0.4304 | 0.1068 | 0.0154 | 3.73E-12 |
| rs2478835 | T | C | 0.4378 | -0.1076 | 0.0152 | 1.43E-12 |
| rs2484294 | A | G | 0.7301 | 0.2854 | 0.0169 | 1.00E-63 |
| rs2493136 | T | C | 0.4109 | 0.2166 | 0.0152 | 4.65E-46 |
| rs2493288 | A | G | 0.1441 | 0.2213 | 0.0219 | 4.35E-24 |
| rs2517938 | C | T | 0.173 | 0.1352 | 0.0204 | 3.35E-11 |
| rs2569882 | C | T | 0.4348 | -0.1087 | 0.0156 | 3.14E-12 |
| rs2586970 | G | A | 0.5634 | 0.1351 | 0.0152 | 6.19E-19 |
| rs2591860 | T | C | 0.3531 | 0.1019 | 0.0157 | 8.76E-11 |
| rs2618647 | G | A | 0.4949 | 0.098 | 0.0151 | 9.01E-11 |
| rs2627316 | G | A | 0.4689 | 0.1382 | 0.0151 | 4.35E-20 |
| rs2643826 | T | C | 0.452 | 0.1866 | 0.0153 | 3.47E-34 |
| rs2649792 | G | T | 0.871 | -0.1258 | 0.0227 | 2.94E-08 |
| rs2681485 | A | G | 0.5971 | 0.2718 | 0.0153 | 4.88E-71 |
| rs2690115 | A | G | 0.4172 | -0.0868 | 0.0152 | 1.18E-08 |
| rs2695258 | T | C | 0.2129 | 0.1189 | 0.0182 | 7.02E-11 |
| rs27300 | T | C | 0.5651 | -0.1177 | 0.0152 | 1.17E-14 |
| rs2731625 | G | A | 0.5277 | 0.0978 | 0.015 | 7.61E-11 |
| rs2744133 | G | A | 0.2742 | -0.1249 | 0.0168 | 1.10E-13 |
| rs2807337 | C | T | 0.6323 | -0.0868 | 0.0155 | 2.12E-08 |
| rs28360984 | G | A | 0.0579 | 0.2627 | 0.0333 | 3.21E-15 |
| rs2836411 | T | C | 0.3469 | 0.1439 | 0.016 | 2.53E-19 |
| rs28377357 | A | G | 0.2903 | -0.101 | 0.0165 | 8.69E-10 |
| rs28429256 | A | G | 0.3343 | 0.1388 | 0.0163 | 1.92E-17 |
| rs2854746 | C | G | 0.4012 | 0.1214 | 0.0156 | 7.06E-15 |
| rs28551743 | A | G | 0.512 | -0.1546 | 0.0151 | 1.34E-24 |
| rs28564120 | G | A | 0.2151 | 0.1292 | 0.0191 | 1.25E-11 |
| rs28590346 | T | A | 0.3406 | 0.1823 | 0.0163 | 4.47E-29 |
| rs28628236 | G | A | 0.4009 | -0.0972 | 0.0154 | 2.57E-10 |
| rs28658085 | A | G | 0.4046 | -0.0894 | 0.0155 | 7.83E-09 |
| rs28661492 | T | C | 0.2041 | -0.1234 | 0.0192 | 1.16E-10 |
| rs28667801 | T | A | 0.4067 | 0.1375 | 0.0156 | 1.10E-18 |
| rs28675079 | A | G | 0.1861 | -0.1283 | 0.0192 | 2.48E-11 |
| rs2877766 | T | G | 0.4227 | -0.0926 | 0.0152 | 1.22E-09 |
| rs2893847 | A | C | 0.1567 | 0.1527 | 0.0206 | 1.34E-13 |
| rs2921604 | C | T | 0.4652 | 0.0839 | 0.0153 | 4.02E-08 |
| rs2926534 | T | C | 0.2702 | -0.1623 | 0.0169 | 7.71E-22 |
| rs2969070 | A | G | 0.6301 | -0.1666 | 0.0156 | 1.69E-26 |
| rs2972579 | C | T | 0.6051 | -0.1096 | 0.0158 | 3.78E-12 |
| rs297690 | G | T | 0.7339 | 0.1019 | 0.0171 | 2.84E-09 |
| rs2978098 | C | A | 0.457 | -0.1426 | 0.0151 | 4.34E-21 |
| rs2997336 | A | G | 0.5356 | -0.0868 | 0.015 | 7.80E-09 |
| rs3013093 | C | T | 0.3959 | -0.0868 | 0.0153 | 1.36E-08 |
| rs301791 | T | A | 0.332 | 0.1085 | 0.0159 | 8.82E-12 |
| rs3027160 | C | T | 0.2292 | -0.1108 | 0.0182 | 1.26E-09 |
| rs3107147 | A | G | 0.6023 | 0.107 | 0.0155 | 5.37E-12 |
| rs3112522 | A | G | 0.465 | 0.1061 | 0.0151 | 2.18E-12 |
| rs311427 | A | G | 0.7166 | 0.1151 | 0.0167 | 5.68E-12 |
| rs3117736 | T | C | 0.2671 | 0.1983 | 0.017 | 1.53E-31 |
| rs3129716 | C | T | 0.1379 | -0.3208 | 0.023 | 2.55E-44 |
| rs3129817 | A | G | 0.1353 | -0.2667 | 0.0232 | 1.14E-30 |
| rs3212303 | G | A | 0.5912 | -0.1292 | 0.0156 | 1.18E-16 |
| rs32490 | C | T | 0.3069 | -0.0908 | 0.0162 | 2.10E-08 |
| rs335170 | C | A | 0.5909 | -0.1144 | 0.0153 | 6.71E-14 |
| rs338526 | T | C | 0.7183 | 0.1044 | 0.0167 | 4.08E-10 |
| rs33916666 | C | T | 0.5487 | -0.108 | 0.0152 | 1.15E-12 |
| rs34039016 | A | G | 0.2663 | 0.1852 | 0.0171 | 2.90E-27 |
| rs34105469 | T | C | 0.3825 | 0.0883 | 0.0156 | 1.51E-08 |
| rs34130368 | T | G | 0.1172 | -0.1821 | 0.0241 | 4.56E-14 |
| rs34163044 | A | C | 0.4191 | 0.1418 | 0.0156 | 8.74E-20 |
| rs34237622 | A | G | 0.1653 | -0.1135 | 0.0206 | 3.72E-08 |
| rs34287081 | T | G | 0.2395 | 0.1163 | 0.0177 | 5.14E-11 |
| rs342989 | G | A | 0.7727 | -0.1392 | 0.0179 | 7.06E-15 |
| rs34413141 | A | T | 0.1825 | -0.177 | 0.0198 | 4.16E-19 |
| rs34430710 | T | A | 0.3233 | 0.1218 | 0.0162 | 5.25E-14 |
| rs34517439 | A | C | 0.1176 | -0.2297 | 0.0246 | 9.01E-21 |
| rs34538877 | T | C | 0.4231 | -0.1078 | 0.0154 | 2.20E-12 |
| rs34547756 | G | T | 0.7397 | -0.1019 | 0.0174 | 4.81E-09 |
| rs34587839 | A | G | 0.1517 | -0.157 | 0.0213 | 1.84E-13 |
| rs34612537 | C | T | 0.2079 | 0.1394 | 0.0186 | 6.51E-14 |
| rs347585 | T | C | 0.7 | 0.1528 | 0.0164 | 9.72E-21 |
| rs34788903 | C | T | 0.1066 | 0.1982 | 0.025 | 2.43E-15 |
| rs35074129 | G | A | 0.1292 | 0.1625 | 0.0227 | 7.80E-13 |
| rs35098301 | C | T | 0.3934 | 0.0996 | 0.0156 | 1.58E-10 |
| rs35213536 | T | G | 0.2462 | 0.1853 | 0.0181 | 1.23E-24 |
| rs35261542 | A | C | 0.2673 | 0.0974 | 0.0169 | 8.37E-09 |
| rs35287509 | C | T | 0.3364 | 0.0906 | 0.0159 | 1.15E-08 |
| rs35443 | C | G | 0.3857 | -0.2543 | 0.0154 | 1.19E-61 |
| rs35667547 | C | G | 0.1208 | 0.1504 | 0.0258 | 5.76E-09 |
| rs35732435 | T | C | 0.3025 | 0.1309 | 0.0164 | 1.30E-15 |
| rs35743822 | C | T | 0.1489 | 0.1483 | 0.0221 | 1.81E-11 |
| rs357489 | A | T | 0.7421 | -0.1376 | 0.0172 | 1.24E-15 |
| rs35761891 | G | A | 0.0466 | 0.2922 | 0.0501 | 5.35E-09 |
| rs35767319 | A | G | 0.6018 | -0.0929 | 0.0153 | 1.33E-09 |
| rs35781150 | G | A | 0.2255 | 0.1223 | 0.0182 | 1.85E-11 |
| rs35783704 | A | G | 0.1018 | -0.2022 | 0.0257 | 3.72E-15 |
| rs35792872 | A | G | 0.4946 | 0.0951 | 0.0152 | 3.52E-10 |
| rs35981664 | T | A | 0.3082 | 0.1603 | 0.0166 | 3.72E-22 |
| rs36022378 | C | T | 0.2 | 0.1738 | 0.019 | 6.65E-20 |
| rs36027301 | T | C | 0.0591 | -0.2329 | 0.0326 | 9.63E-13 |
| rs36049616 | T | G | 0.0315 | 0.3133 | 0.0438 | 8.29E-13 |
| rs36069097 | A | G | 0.2655 | -0.2352 | 0.0174 | 1.36E-41 |
| rs3731851 | T | A | 0.1981 | 0.1163 | 0.0193 | 1.53E-09 |
| rs3735318 | A | G | 0.5389 | -0.0933 | 0.0149 | 4.36E-10 |
| rs3735533 | C | T | 0.9252 | 0.4482 | 0.0286 | 1.64E-55 |
| rs3740237 | C | G | 0.1413 | 0.141 | 0.0221 | 1.79E-10 |
| rs3741983 | T | C | 0.1826 | -0.2788 | 0.0198 | 7.04E-45 |
| rs3742485 | G | A | 0.2166 | 0.1162 | 0.0183 | 2.40E-10 |
| rs3744010 | A | G | 0.247 | -0.1331 | 0.0175 | 2.97E-14 |
| rs3745688 | T | C | 0.0742 | 0.2285 | 0.0297 | 1.37E-14 |
| rs3746467 | C | T | 0.4669 | -0.089 | 0.0151 | 3.42E-09 |
| rs3750944 | A | G | 0.5558 | 0.0891 | 0.0152 | 4.24E-09 |
| rs3757914 | A | G | 0.715 | -0.1139 | 0.0169 | 1.58E-11 |
| rs3765618 | G | C | 0.0875 | -0.1802 | 0.0273 | 3.87E-11 |
| rs3766614 | A | G | 0.3659 | 0.0931 | 0.0156 | 2.66E-09 |
| rs3766683 | A | G | 0.5632 | -0.1006 | 0.015 | 2.30E-11 |
| rs3776299 | A | G | 0.4558 | 0.1105 | 0.0152 | 3.15E-13 |
| rs3789367 | T | A | 0.3883 | -0.0908 | 0.0154 | 3.67E-09 |
| rs3796581 | G | A | 0.1865 | -0.2287 | 0.0192 | 8.71E-33 |
| rs3807101 | T | C | 0.1227 | -0.1616 | 0.023 | 1.98E-12 |
| rs3808868 | G | T | 0.491 | 0.0891 | 0.0151 | 3.20E-09 |
| rs3814415 | G | A | 0.1559 | 0.1158 | 0.0209 | 3.09E-08 |
| rs3821843 | A | G | 0.682 | 0.1652 | 0.0167 | 5.10E-23 |
| rs3889391 | A | G | 0.4292 | -0.0878 | 0.0151 | 5.78E-09 |
| rs3916033 | T | C | 0.558 | -0.0994 | 0.0159 | 4.11E-10 |
| rs3918226 | T | C | 0.0808 | 0.5519 | 0.0289 | 1.42E-81 |
| rs39281 | G | A | 0.5244 | -0.1316 | 0.0153 | 7.71E-18 |
| rs4007357 | A | G | 0.6835 | 0.0894 | 0.0163 | 3.85E-08 |
| rs4077158 | C | T | 0.5278 | 0.1833 | 0.015 | 1.83E-34 |
| rs4129585 | C | A | 0.5595 | -0.087 | 0.0151 | 7.97E-09 |
| rs4244200 | C | G | 0.2799 | -0.1108 | 0.0168 | 4.00E-11 |
| rs4264931 | A | G | 0.4173 | 0.1035 | 0.0151 | 8.00E-12 |
| rs4279157 | A | T | 0.6789 | -0.1319 | 0.0161 | 2.54E-16 |
| rs4306343 | T | A | 0.7204 | 0.2708 | 0.0166 | 1.27E-59 |
| rs4315061 | C | T | 0.4954 | -0.1255 | 0.0173 | 3.88E-13 |
| rs4354288 | A | T | 0.6086 | 0.0888 | 0.0153 | 6.85E-09 |
| rs4357173 | A | G | 0.7855 | -0.1188 | 0.0183 | 8.36E-11 |
| rs4362428 | A | C | 0.407 | -0.1061 | 0.0153 | 4.02E-12 |
| rs4411245 | A | G | 0.2943 | 0.0952 | 0.0164 | 6.89E-09 |
| rs4499560 | T | A | 0.6808 | 0.0989 | 0.0163 | 1.17E-09 |
| rs4507656 | G | C | 0.3041 | 0.1352 | 0.0171 | 2.59E-15 |
| rs451367 | C | T | 0.8035 | -0.114 | 0.0198 | 8.25E-09 |
| rs45474499 | T | C | 0.0472 | 0.3425 | 0.0359 | 1.34E-21 |
| rs4556017 | T | C | 0.8558 | -0.1563 | 0.0222 | 1.88E-12 |
| rs4576073 | G | A | 0.5295 | -0.0903 | 0.0157 | 8.88E-09 |
| rs4587807 | A | G | 0.5114 | -0.0901 | 0.0149 | 1.59E-09 |
| rs4615669 | G | A | 0.4391 | 0.1123 | 0.0151 | 1.03E-13 |
| rs4634143 | C | T | 0.7021 | -0.1081 | 0.0165 | 5.21E-11 |
| rs4646534 | G | A | 0.053 | -0.3006 | 0.0341 | 1.30E-18 |
| rs4666006 | T | G | 0.4731 | 0.0825 | 0.0149 | 3.37E-08 |
| rs4670548 | G | A | 0.6795 | -0.101 | 0.016 | 2.49E-10 |
| rs4675297 | G | A | 0.329 | 0.1231 | 0.0162 | 3.07E-14 |
| rs4675682 | C | T | 0.4576 | 0.1271 | 0.0151 | 4.54E-17 |
| rs4679113 | A | T | 0.6336 | -0.1184 | 0.0156 | 3.51E-14 |
| rs4693973 | A | T | 0.2243 | 0.1117 | 0.0182 | 7.55E-10 |
| rs4699835 | A | G | 0.4619 | 0.0961 | 0.0151 | 2.15E-10 |
| rs4714224 | C | G | 0.2767 | -0.1347 | 0.0174 | 8.38E-15 |
| rs4723948 | C | T | 0.2219 | 0.1034 | 0.0182 | 1.45E-08 |
| rs4726006 | A | G | 0.2534 | 0.1271 | 0.0174 | 2.60E-13 |
| rs4729301 | T | C | 0.5787 | 0.1411 | 0.0152 | 1.30E-20 |
| rs4739832 | C | A | 0.4134 | -0.1209 | 0.0153 | 2.78E-15 |
| rs4746139 | C | A | 0.1417 | 0.1654 | 0.0217 | 2.58E-14 |
| rs4766994 | C | T | 0.2091 | -0.1073 | 0.0185 | 6.54E-09 |
| rs4793077 | T | C | 0.7422 | 0.0961 | 0.0174 | 3.12E-08 |
| rs4805682 | A | G | 0.3828 | 0.0996 | 0.0154 | 1.02E-10 |
| rs4835377 | G | A | 0.8333 | -0.1135 | 0.0203 | 2.17E-08 |
| rs4850047 | C | T | 0.856 | 0.1324 | 0.022 | 1.72E-09 |
| rs4894808 | C | G | 0.4075 | 0.1184 | 0.0162 | 3.15E-13 |
| rs4903064 | C | T | 0.2366 | -0.1454 | 0.0177 | 2.32E-16 |
| rs4908670 | T | A | 0.3959 | 0.0929 | 0.0154 | 1.73E-09 |
| rs4909314 | A | T | 0.392 | 0.1151 | 0.0153 | 5.52E-14 |
| rs4917911 | A | G | 0.8882 | 0.3003 | 0.0238 | 2.17E-36 |
| rs4925247 | T | C | 0.5452 | -0.095 | 0.0163 | 6.12E-09 |
| rs4926499 | C | G | 0.825 | 0.1724 | 0.0223 | 1.21E-14 |
| rs4926923 | C | T | 0.089 | -0.159 | 0.0268 | 2.96E-09 |
| rs4936099 | A | C | 0.6016 | 0.1673 | 0.0155 | 2.72E-27 |
| rs4940576 | C | T | 0.7594 | 0.1069 | 0.0176 | 1.39E-09 |
| rs4948550 | C | T | 0.7286 | 0.1054 | 0.0168 | 3.18E-10 |
| rs4948643 | C | T | 0.7172 | -0.1447 | 0.0167 | 4.83E-18 |
| rs4963772 | A | G | 0.1503 | -0.1302 | 0.0211 | 6.18E-10 |
| rs4972540 | G | A | 0.0836 | 0.1596 | 0.0272 | 4.33E-09 |
| rs4973930 | C | A | 0.5227 | 0.091 | 0.015 | 1.40E-09 |
| rs4987082 | C | T | 0.4322 | 0.2066 | 0.0151 | 1.60E-42 |
| rs504691 | A | C | 0.4019 | -0.1014 | 0.0154 | 4.18E-11 |
| rs509564 | T | C | 0.2269 | 0.1045 | 0.018 | 6.36E-09 |
| rs513563 | T | C | 0.6474 | -0.095 | 0.0157 | 1.63E-09 |
| rs525271 | T | C | 0.293 | 0.1043 | 0.0165 | 2.50E-10 |
| rs538180 | A | T | 0.4173 | -0.0867 | 0.0152 | 1.24E-08 |
| rs544067 | C | T | 0.3224 | -0.0921 | 0.016 | 9.06E-09 |
| rs555075 | T | C | 0.4505 | -0.0871 | 0.0152 | 8.95E-09 |
| rs55641580 | T | C | 0.1258 | 0.1499 | 0.0229 | 5.32E-11 |
| rs55683214 | T | G | 0.2536 | -0.1383 | 0.0172 | 9.71E-16 |
| rs55684003 | G | A | 0.302 | -0.1201 | 0.0163 | 1.98E-13 |
| rs55706574 | A | G | 0.6196 | -0.0968 | 0.0166 | 5.23E-09 |
| rs55710016 | A | G | 0.4709 | 0.1025 | 0.015 | 8.05E-12 |
| rs55889649 | A | G | 0.4059 | 0.1212 | 0.0152 | 1.72E-15 |
| rs55936760 | G | A | 0.4407 | -0.0839 | 0.0153 | 4.08E-08 |
| rs55938136 | G | A | 0.2348 | -0.1425 | 0.0224 | 1.90E-10 |
| rs56228561 | T | G | 0.0667 | 0.1921 | 0.0308 | 4.25E-10 |
| rs56256111 | A | G | 0.1425 | 0.1646 | 0.023 | 8.72E-13 |
| rs56258383 | A | G | 0.2886 | -0.1013 | 0.0167 | 1.18E-09 |
| rs56351548 | C | T | 0.1271 | 0.1489 | 0.0227 | 5.94E-11 |
| rs56388530 | T | C | 0.7618 | 0.2225 | 0.0177 | 2.39E-36 |
| rs56389811 | T | C | 0.2329 | -0.1715 | 0.0178 | 5.65E-22 |
| rs56401299 | T | C | 0.3519 | 0.1008 | 0.0162 | 5.11E-10 |
| rs568032 | G | A | 0.9412 | 0.2194 | 0.0335 | 5.56E-11 |
| rs569550 | G | T | 0.3958 | 0.239 | 0.0157 | 1.94E-52 |
| rs57060554 | G | A | 0.2576 | -0.1173 | 0.0172 | 1.02E-11 |
| rs57067187 | C | T | 0.3722 | -0.1135 | 0.0165 | 5.26E-12 |
| rs57091267 | A | G | 0.2014 | -0.1689 | 0.0192 | 1.42E-18 |
| rs57327054 | T | C | 0.3086 | -0.0903 | 0.0165 | 4.40E-08 |
| rs57503539 | A | G | 0.2098 | -0.1182 | 0.0188 | 3.42E-10 |
| rs57541197 | A | G | 0.1408 | -0.4301 | 0.0215 | 3.63E-89 |
| rs57748895 | T | A | 0.0183 | 0.5855 | 0.0574 | 1.93E-24 |
| rs57786342 | A | G | 0.206 | 0.1123 | 0.0187 | 1.78E-09 |
| rs57989773 | C | T | 0.2454 | -0.1228 | 0.0184 | 2.49E-11 |
| rs58580703 | A | C | 0.0596 | -0.2132 | 0.0319 | 2.38E-11 |
| rs58609847 | T | C | 0.3972 | -0.0901 | 0.0154 | 4.43E-09 |
| rs58847484 | C | T | 0.211 | -0.1305 | 0.0188 | 3.81E-12 |
| rs58854324 | C | T | 0.1563 | 0.1333 | 0.0209 | 1.62E-10 |
| rs59333122 | A | C | 0.2781 | 0.1074 | 0.0173 | 5.96E-10 |
| rs59400568 | G | A | 0.1964 | -0.1608 | 0.0192 | 5.02E-17 |
| rs59434918 | C | T | 0.5387 | 0.0858 | 0.0155 | 3.09E-08 |
| rs598682 | G | A | 0.75 | 0.1116 | 0.0172 | 9.81E-11 |
| rs5992929 | T | C | 0.2833 | 0.1412 | 0.0167 | 3.37E-17 |
| rs59971314 | C | G | 0.2846 | 0.1191 | 0.0168 | 1.31E-12 |
| rs600951 | G | A | 0.5451 | -0.0901 | 0.0153 | 3.75E-09 |
| rs601338 | A | G | 0.4926 | 0.1169 | 0.0151 | 9.76E-15 |
| rs6021247 | A | G | 0.5315 | 0.1178 | 0.015 | 3.78E-15 |
| rs60218157 | C | A | 0.0266 | 0.3319 | 0.0487 | 9.22E-12 |
| rs6026578 | G | C | 0.6264 | 0.1065 | 0.0158 | 1.86E-11 |
| rs6039211 | G | A | 0.3652 | -0.1478 | 0.0156 | 3.38E-21 |
| rs6040421 | G | A | 0.5045 | -0.0974 | 0.015 | 9.13E-11 |
| rs6046137 | G | A | 0.258 | -0.1154 | 0.0172 | 1.82E-11 |
| rs604723 | C | T | 0.7231 | 0.3308 | 0.0169 | 4.09E-85 |
| rs6054139 | A | G | 0.6085 | 0.1181 | 0.0153 | 1.21E-14 |
| rs6060262 | C | A | 0.1636 | -0.1748 | 0.0204 | 1.20E-17 |
| rs6122713 | G | C | 0.3116 | -0.1228 | 0.0163 | 4.85E-14 |
| rs6130553 | T | C | 0.1336 | 0.1836 | 0.0219 | 5.67E-17 |
| rs6131281 | T | C | 0.3988 | -0.12 | 0.0153 | 4.51E-15 |
| rs613808 | G | A | 0.7054 | -0.1259 | 0.0167 | 4.03E-14 |
| rs61789369 | G | A | 0.0443 | 0.2686 | 0.0378 | 1.27E-12 |
| rs61879810 | G | A | 0.8499 | -0.1425 | 0.0211 | 1.48E-11 |
| rs61909958 | G | C | 0.1879 | -0.1233 | 0.0199 | 6.11E-10 |
| rs61910264 | T | C | 0.4575 | 0.0873 | 0.015 | 6.19E-09 |
| rs61911503 | A | G | 0.1223 | 0.1771 | 0.0232 | 2.43E-14 |
| rs61912332 | C | T | 0.4986 | -0.1008 | 0.0153 | 5.11E-11 |
| rs62004794 | G | A | 0.5591 | 0.0857 | 0.0151 | 1.37E-08 |
| rs62012629 | A | C | 0.267 | -0.1762 | 0.0178 | 3.95E-23 |
| rs62052380 | T | C | 0.1364 | 0.1568 | 0.0282 | 2.82E-08 |
| rs62155750 | G | A | 0.3041 | 0.1926 | 0.0169 | 3.36E-30 |
| rs62158169 | T | C | 0.2171 | -0.1289 | 0.0183 | 2.03E-12 |
| rs62229372 | T | C | 0.1249 | 0.1385 | 0.024 | 7.75E-09 |
| rs62301873 | G | A | 0.1058 | 0.1453 | 0.0245 | 3.06E-09 |
| rs62370646 | C | A | 0.1878 | -0.1188 | 0.0193 | 7.97E-10 |
| rs62406520 | G | A | 0.0979 | -0.1469 | 0.0266 | 3.17E-08 |
| rs62413470 | A | G | 0.1509 | -0.1635 | 0.021 | 6.50E-15 |
| rs62503324 | T | C | 0.2422 | 0.1876 | 0.0175 | 8.56E-27 |
| rs6271 | T | C | 0.0726 | -0.3777 | 0.0304 | 2.22E-35 |
| rs627524 | C | A | 0.4849 | 0.0853 | 0.0152 | 2.00E-08 |
| rs629864 | T | C | 0.6519 | -0.0959 | 0.016 | 2.03E-09 |
| rs638310 | A | G | 0.6604 | 0.1321 | 0.0159 | 9.24E-17 |
| rs6442105 | G | A | 0.6717 | 0.2323 | 0.016 | 8.65E-48 |
| rs6445818 | A | C | 0.3147 | -0.1675 | 0.0162 | 4.23E-25 |
| rs6464165 | C | T | 0.2821 | 0.203 | 0.0168 | 9.93E-34 |
| rs647451 | T | C | 0.4676 | -0.091 | 0.0153 | 2.59E-09 |
| rs6487076 | G | A | 0.2223 | -0.1657 | 0.0181 | 6.26E-20 |
| rs6497759 | A | G | 0.1931 | -0.1116 | 0.019 | 4.18E-09 |
| rs6503732 | C | T | 0.3668 | 0.088 | 0.016 | 3.62E-08 |
| rs650724 | A | G | 0.0952 | -0.1913 | 0.0264 | 4.70E-13 |
| rs6511291 | C | T | 0.5617 | 0.0964 | 0.0154 | 4.33E-10 |
| rs6539344 | T | G | 0.5806 | 0.0864 | 0.0156 | 2.78E-08 |
| rs6541771 | A | G | 0.4367 | -0.0941 | 0.0154 | 9.86E-10 |
| rs6556384 | A | C | 0.8102 | -0.1346 | 0.0193 | 3.02E-12 |
| rs6587216 | C | G | 0.8073 | 0.1404 | 0.0195 | 6.79E-13 |
| rs6600228 | C | A | 0.3645 | 0.109 | 0.0158 | 5.38E-12 |
| rs6602177 | T | C | 0.7059 | -0.1096 | 0.0183 | 1.98E-09 |
| rs6669446 | C | T | 0.4214 | -0.0889 | 0.0151 | 4.29E-09 |
| rs6671476 | A | T | 0.7332 | -0.0936 | 0.0169 | 3.34E-08 |
| rs6680947 | G | A | 0.5347 | -0.1064 | 0.015 | 1.51E-12 |
| rs668459 | T | C | 0.5866 | -0.1217 | 0.0151 | 9.12E-16 |
| rs6686889 | T | C | 0.2537 | 0.1726 | 0.0173 | 1.90E-23 |
| rs6688009 | T | C | 0.1599 | 0.1199 | 0.0206 | 5.42E-09 |
| rs66887589 | C | T | 0.4783 | 0.1432 | 0.0151 | 2.71E-21 |
| rs6690771 | C | G | 0.7346 | -0.1282 | 0.0169 | 3.27E-14 |
| rs6699618 | G | C | 0.1597 | -0.4744 | 0.0204 | 7.61E-120 |
| rs67323263 | C | T | 0.2044 | 0.131 | 0.0186 | 1.81E-12 |
| rs6742187 | T | C | 0.5766 | -0.096 | 0.0153 | 3.18E-10 |
| rs6748833 | G | T | 0.5817 | -0.0889 | 0.0153 | 5.84E-09 |
| rs6779368 | G | A | 0.3434 | 0.162 | 0.0159 | 2.99E-24 |
| rs67909753 | A | G | 0.29 | -0.1961 | 0.0165 | 1.77E-32 |
| rs6795735 | T | C | 0.4116 | -0.1473 | 0.0171 | 7.53E-18 |
| rs6805393 | A | G | 0.5081 | -0.0826 | 0.0149 | 3.27E-08 |
| rs6807945 | T | C | 0.836 | -0.1643 | 0.0204 | 9.51E-16 |
| rs68085857 | T | C | 0.2347 | 0.1831 | 0.0177 | 3.96E-25 |
| rs68192516 | G | T | 0.3028 | 0.2393 | 0.0163 | 5.58E-49 |
| rs6822301 | G | A | 0.1986 | 0.1081 | 0.0192 | 1.73E-08 |
| rs6838416 | A | G | 0.6884 | -0.1259 | 0.0162 | 9.13E-15 |
| rs6867167 | C | A | 0.6977 | 0.1135 | 0.0164 | 5.09E-12 |
| rs687914 | T | G | 0.2535 | 0.1564 | 0.0176 | 6.75E-19 |
| rs688540 | A | G | 0.8657 | 0.1324 | 0.0226 | 4.72E-09 |
| rs6887553 | G | A | 0.05 | -0.2242 | 0.0395 | 1.36E-08 |
| rs6892983 | A | C | 0.402 | 0.1802 | 0.0152 | 3.19E-32 |
| rs6905288 | A | G | 0.5677 | 0.1537 | 0.0153 | 1.23E-23 |
| rs6914091 | T | C | 0.427 | -0.0898 | 0.0151 | 2.57E-09 |
| rs6920788 | T | C | 0.7168 | 0.0951 | 0.0168 | 1.52E-08 |
| rs693974 | T | C | 0.6044 | -0.1723 | 0.0154 | 4.46E-29 |
| rs6961048 | G | C | 0.1044 | 0.2766 | 0.0246 | 2.90E-29 |
| rs6961414 | A | G | 0.4813 | -0.0869 | 0.015 | 6.89E-09 |
| rs6978112 | T | C | 0.4131 | 0.1019 | 0.0154 | 3.61E-11 |
| rs6990531 | G | T | 0.2475 | -0.113 | 0.0175 | 1.04E-10 |
| rs6999850 | T | A | 0.1888 | 0.112 | 0.0194 | 8.44E-09 |
| rs7014841 | A | C | 0.3599 | -0.0904 | 0.0158 | 9.74E-09 |
| rs7020564 | T | A | 0.3025 | 0.114 | 0.0168 | 1.21E-11 |
| rs705696 | A | G | 0.3369 | -0.0965 | 0.0159 | 1.21E-09 |
| rs706160 | A | G | 0.43 | -0.118 | 0.0153 | 1.31E-14 |
| rs7082671 | A | G | 0.017 | 0.4428 | 0.059 | 5.95E-14 |
| rs7093678 | C | T | 0.5245 | -0.0834 | 0.0151 | 3.48E-08 |
| rs7095584 | A | G | 0.618 | 0.0865 | 0.0155 | 2.52E-08 |
| rs7098414 | C | A | 0.7317 | -0.0993 | 0.017 | 5.39E-09 |
| rs7105402 | C | A | 0.1421 | -0.2058 | 0.0216 | 1.81E-21 |
| rs7109016 | T | A | 0.7037 | 0.1429 | 0.0164 | 2.71E-18 |
| rs7123006 | G | A | 0.4355 | 0.0877 | 0.0151 | 6.31E-09 |
| rs7123653 | C | A | 0.4081 | -0.0881 | 0.0154 | 1.11E-08 |
| rs71316245 | G | A | 0.3837 | -0.0871 | 0.0155 | 1.92E-08 |
| rs7134060 | A | G | 0.4462 | -0.0967 | 0.015 | 1.30E-10 |
| rs7134440 | T | C | 0.0825 | 0.202 | 0.0281 | 6.17E-13 |
| rs7134677 | T | C | 0.2959 | -0.1692 | 0.0166 | 2.62E-24 |
| rs7137749 | C | T | 0.6304 | -0.1351 | 0.0156 | 6.09E-18 |
| rs7137828 | T | C | 0.5172 | -0.4348 | 0.0151 | 7.03E-182 |
| rs7147275 | A | G | 0.134 | -0.1366 | 0.0248 | 3.71E-08 |
| rs7155504 | C | T | 0.0879 | -0.2033 | 0.0272 | 8.47E-14 |
| rs7172082 | G | A | 0.1952 | 0.1202 | 0.0193 | 5.17E-10 |
| rs7174546 | G | A | 0.6666 | -0.1645 | 0.016 | 6.23E-25 |
| rs7174732 | A | C | 0.7212 | -0.1425 | 0.0169 | 3.43E-17 |
| rs7174977 | T | A | 0.637 | 0.0911 | 0.0158 | 8.15E-09 |
| rs7193889 | T | G | 0.5542 | 0.0892 | 0.0152 | 4.17E-09 |
| rs7200353 | A | G | 0.6116 | -0.1031 | 0.0157 | 5.71E-11 |
| rs7203157 | C | T | 0.5909 | 0.1127 | 0.0152 | 1.30E-13 |
| rs720782 | T | C | 0.4426 | 0.0853 | 0.0151 | 1.56E-08 |
| rs7221807 | C | T | 0.4229 | 0.1353 | 0.0151 | 3.89E-19 |
| rs7224296 | A | G | 0.7265 | 0.1289 | 0.0192 | 1.71E-11 |
| rs7227658 | A | T | 0.0793 | 0.2254 | 0.0314 | 7.05E-13 |
| rs722783 | A | G | 0.2206 | -0.204 | 0.018 | 9.70E-30 |
| rs7246865 | A | G | 0.2677 | 0.1888 | 0.0173 | 7.27E-28 |
| rs7257694 | T | C | 0.3964 | 0.1785 | 0.0155 | 1.48E-30 |
| rs72613226 | G | A | 0.1299 | 0.1672 | 0.024 | 3.27E-12 |
| rs7265695 | C | T | 0.1998 | -0.1926 | 0.0191 | 5.99E-24 |
| rs72693377 | T | C | 0.0433 | -0.2563 | 0.0397 | 1.13E-10 |
| rs72771851 | A | C | 0.0422 | 0.2319 | 0.0382 | 1.25E-09 |
| rs72772056 | A | G | 0.1501 | 0.1162 | 0.0212 | 4.25E-08 |
| rs72788766 | A | G | 0.3595 | -0.0941 | 0.0156 | 1.68E-09 |
| rs72814384 | A | T | 0.1718 | -0.137 | 0.0199 | 5.61E-12 |
| rs72831853 | T | A | 0.2562 | -0.1162 | 0.0171 | 1.14E-11 |
| rs72842207 | T | C | 0.2132 | -0.211 | 0.0183 | 6.76E-31 |
| rs72851229 | C | G | 0.1739 | -0.1174 | 0.0201 | 5.36E-09 |
| rs72854462 | G | A | 0.2464 | 0.2217 | 0.0174 | 2.38E-37 |
| rs72936986 | C | A | 0.2808 | -0.096 | 0.0167 | 8.23E-09 |
| rs72976750 | C | T | 0.139 | 0.1517 | 0.0218 | 3.45E-12 |
| rs73033340 | G | A | 0.0346 | -0.4841 | 0.0472 | 1.01E-24 |
| rs73113959 | T | C | 0.2713 | 0.0939 | 0.0169 | 2.65E-08 |
| rs7316428 | C | G | 0.2652 | -0.1431 | 0.0174 | 2.00E-16 |
| rs7321585 | C | G | 0.2612 | 0.0962 | 0.0171 | 1.88E-08 |
| rs73231988 | A | G | 0.1166 | 0.1352 | 0.0238 | 1.45E-08 |
| rs73280613 | A | G | 0.0559 | -0.1911 | 0.033 | 7.08E-09 |
| rs7331882 | A | G | 0.2355 | 0.1534 | 0.0177 | 4.42E-18 |
| rs7350752 | A | G | 0.124 | -0.1465 | 0.0261 | 1.89E-08 |
| rs73530785 | G | A | 0.0772 | 0.2467 | 0.0324 | 2.82E-14 |
| rs73563812 | T | G | 0.2378 | -0.1684 | 0.0176 | 9.86E-22 |
| rs7358352 | C | T | 0.144 | -0.188 | 0.0217 | 4.34E-18 |
| rs73954926 | G | T | 0.0633 | 0.1719 | 0.0315 | 4.67E-08 |
| rs74048200 | G | A | 0.0859 | 0.1859 | 0.0281 | 3.66E-11 |
| rs7407 | T | C | 0.5065 | -0.1292 | 0.0177 | 3.32E-13 |
| rs7427249 | A | G | 0.5802 | -0.0879 | 0.0152 | 7.00E-09 |
| rs74439044 | C | T | 0.0979 | 0.3399 | 0.0254 | 1.05E-40 |
| rs7454297 | A | G | 0.8891 | -0.1413 | 0.024 | 4.09E-09 |
| rs7461159 | A | T | 0.7326 | 0.1001 | 0.017 | 3.90E-09 |
| rs74734425 | T | C | 0.046 | 0.3842 | 0.0362 | 2.55E-26 |
| rs7491960 | T | C | 0.4874 | -0.1101 | 0.0156 | 1.66E-12 |
| rs7494379 | T | C | 0.3082 | 0.1088 | 0.0164 | 3.01E-11 |
| rs7500448 | G | A | 0.2519 | 0.1272 | 0.0177 | 6.51E-13 |
| rs75054140 | T | C | 0.028 | 0.3276 | 0.0558 | 4.34E-09 |
| rs7516453 | G | C | 0.2764 | -0.1002 | 0.0168 | 2.63E-09 |
| rs751984 | C | T | 0.1181 | -0.3398 | 0.0238 | 2.48E-46 |
| rs752092 | G | A | 0.3315 | 0.0906 | 0.016 | 1.59E-08 |
| rs7524019 | T | C | 0.4872 | 0.0998 | 0.0156 | 1.49E-10 |
| rs75488436 | T | C | 0.1451 | 0.1609 | 0.0213 | 4.22E-14 |
| rs75507123 | T | G | 0.1286 | -0.1416 | 0.0226 | 3.81E-10 |
| rs75511781 | G | A | 0.0441 | 0.3368 | 0.0392 | 8.96E-18 |
| rs755251 | G | A | 0.2488 | 0.1172 | 0.0174 | 1.80E-11 |
| rs7553422 | C | T | 0.5878 | 0.1081 | 0.0152 | 1.30E-12 |
| rs7562 | C | T | 0.4785 | -0.0888 | 0.0151 | 4.27E-09 |
| rs7569128 | A | C | 0.8189 | 0.1855 | 0.0196 | 3.39E-21 |
| rs7575189 | A | G | 0.5812 | 0.1056 | 0.0152 | 3.69E-12 |
| rs7581427 | C | T | 0.3186 | -0.0884 | 0.016 | 3.60E-08 |
| rs758445 | T | C | 0.0994 | -0.1626 | 0.0256 | 2.07E-10 |
| rs7605335 | A | G | 0.4199 | -0.1638 | 0.0152 | 3.29E-27 |
| rs7606205 | C | A | 0.2981 | 0.1193 | 0.0166 | 5.87E-13 |
| rs76073047 | A | G | 0.0501 | 0.2274 | 0.0365 | 4.77E-10 |
| rs7611674 | G | T | 0.1963 | -0.1319 | 0.0194 | 1.13E-11 |
| rs7612045 | A | C | 0.3307 | 0.1135 | 0.0161 | 1.61E-12 |
| rs7615227 | T | G | 0.1304 | 0.1322 | 0.0229 | 7.27E-09 |
| rs76326501 | C | A | 0.0897 | -0.3637 | 0.0267 | 3.25E-42 |
| rs76452347 | T | C | 0.2054 | -0.1914 | 0.0194 | 6.41E-23 |
| rs76704686 | T | C | 0.504 | 0.0961 | 0.0151 | 1.86E-10 |
| rs7670707 | G | A | 0.0196 | 0.3789 | 0.0577 | 5.22E-11 |
| rs7671332 | C | T | 0.0394 | 0.2331 | 0.0397 | 4.27E-09 |
| rs7674762 | A | G | 0.4383 | -0.1298 | 0.0153 | 2.34E-17 |
| rs767717 | C | T | 0.629 | -0.114 | 0.0156 | 2.80E-13 |
| rs7694643 | A | G | 0.6452 | -0.1115 | 0.0156 | 9.72E-13 |
| rs76954792 | T | C | 0.232 | 0.1236 | 0.018 | 6.06E-12 |
| rs77062257 | T | A | 0.2257 | 0.1543 | 0.0264 | 4.97E-09 |
| rs77193180 | C | T | 0.0248 | -0.2844 | 0.051 | 2.51E-08 |
| rs7725317 | C | T | 0.0817 | 0.1874 | 0.033 | 1.35E-08 |
| rs7726162 | C | A | 0.4973 | -0.0962 | 0.0153 | 3.67E-10 |
| rs7744902 | A | G | 0.0778 | -0.2796 | 0.029 | 5.96E-22 |
| rs7753558 | A | C | 0.6345 | 0.137 | 0.0158 | 3.46E-18 |
| rs7753695 | T | C | 0.4383 | 0.0941 | 0.0152 | 5.59E-10 |
| rs7765526 | G | A | 0.534 | -0.0908 | 0.0153 | 2.74E-09 |
| rs7781924 | A | C | 0.5076 | -0.1089 | 0.0151 | 5.31E-13 |
| rs7788746 | T | G | 0.6691 | -0.1455 | 0.016 | 1.07E-19 |
| rs77924615 | A | G | 0.1988 | -0.2551 | 0.0194 | 2.45E-39 |
| rs78013255 | C | T | 0.1908 | -0.2114 | 0.0191 | 2.21E-28 |
| rs78151625 | C | T | 0.1649 | 0.152 | 0.0203 | 6.10E-14 |
| rs7830317 | C | A | 0.5913 | -0.1718 | 0.0154 | 5.37E-29 |
| rs7834533 | T | C | 0.4994 | -0.0818 | 0.0149 | 4.26E-08 |
| rs78381888 | G | A | 0.1833 | 0.1645 | 0.0203 | 5.07E-16 |
| rs7843475 | G | C | 0.2598 | 0.113 | 0.0173 | 5.91E-11 |
| rs78474310 | G | A | 0.044 | 0.2437 | 0.0369 | 3.90E-11 |
| rs78550103 | A | G | 0.1486 | -0.1491 | 0.0216 | 5.36E-12 |
| rs78554005 | T | G | 0.0875 | -0.1911 | 0.0277 | 5.48E-12 |
| rs78648104 | C | T | 0.0894 | 0.1897 | 0.0269 | 1.70E-12 |
| rs78843689 | T | A | 0.0761 | -0.1632 | 0.0286 | 1.12E-08 |
| rs78953748 | G | T | 0.1118 | 0.4487 | 0.024 | 3.77E-78 |
| rs7918084 | T | C | 0.5507 | 0.085 | 0.0152 | 2.25E-08 |
| rs7926335 | T | C | 0.2699 | 0.1586 | 0.0168 | 4.37E-21 |
| rs7927515 | A | C | 0.344 | 0.1052 | 0.0161 | 5.92E-11 |
| rs7927898 | C | T | 0.7183 | 0.131 | 0.0167 | 3.92E-15 |
| rs7927966 | T | C | 0.2417 | -0.133 | 0.0175 | 3.45E-14 |
| rs79375472 | C | A | 0.281 | -0.1202 | 0.0171 | 1.87E-12 |
| rs7959649 | C | T | 0.7553 | -0.1036 | 0.0175 | 3.31E-09 |
| rs79621605 | C | T | 0.0358 | -0.3041 | 0.0412 | 1.62E-13 |
| rs79692989 | T | C | 0.1008 | -0.2029 | 0.0252 | 7.32E-16 |
| rs7975989 | A | G | 0.4979 | -0.0906 | 0.015 | 1.49E-09 |
| rs798538 | C | T | 0.7187 | 0.117 | 0.0168 | 2.88E-12 |
| rs7988232 | A | G | 0.414 | 0.0878 | 0.0154 | 1.15E-08 |
| rs7992292 | A | G | 0.8232 | 0.1155 | 0.0199 | 6.47E-09 |
| rs80045342 | A | G | 0.0713 | -0.164 | 0.0297 | 3.33E-08 |
| rs8005445 | G | A | 0.3364 | 0.0936 | 0.016 | 4.73E-09 |
| rs8008285 | C | T | 0.148 | -0.1177 | 0.0215 | 4.40E-08 |
| rs8014182 | T | C | 0.1324 | -0.1987 | 0.0224 | 6.38E-19 |
| rs8027450 | T | C | 0.3068 | 0.345 | 0.0175 | 9.68E-87 |
| rs8039472 | G | A | 0.5169 | -0.1071 | 0.015 | 9.06E-13 |
| rs8056413 | T | G | 0.5994 | -0.0934 | 0.0155 | 1.75E-09 |
| rs8058961 | A | G | 0.2722 | 0.1426 | 0.0169 | 2.83E-17 |
| rs8068804 | A | G | 0.3243 | 0.1289 | 0.016 | 7.38E-16 |
| rs8077276 | A | G | 0.6206 | -0.1568 | 0.0156 | 1.22E-23 |
| rs8084423 | G | A | 0.5995 | 0.0893 | 0.0154 | 7.05E-09 |
| rs8107974 | T | A | 0.0772 | 0.2553 | 0.0284 | 2.65E-19 |
| rs8128234 | T | C | 0.2113 | 0.102 | 0.0185 | 3.56E-08 |
| rs824522 | A | G | 0.2133 | 0.1563 | 0.0184 | 1.99E-17 |
| rs848309 | C | T | 0.5708 | 0.1207 | 0.0151 | 1.36E-15 |
| rs871606 | C | T | 0.1059 | 0.1653 | 0.0249 | 2.99E-11 |
| rs875106 | A | G | 0.5204 | -0.1173 | 0.015 | 6.12E-15 |
| rs882624 | T | C | 0.3287 | -0.1526 | 0.0162 | 3.85E-21 |
| rs896312 | C | T | 0.3321 | -0.1259 | 0.0161 | 5.25E-15 |
| rs908951 | T | C | 0.4422 | -0.2003 | 0.0156 | 9.99E-38 |
| rs927315 | T | C | 0.4724 | 0.0878 | 0.0152 | 7.92E-09 |
| rs9286351 | G | A | 0.4207 | 0.1438 | 0.0154 | 7.86E-21 |
| rs9291932 | C | T | 0.2733 | 0.1091 | 0.0168 | 9.48E-11 |
| rs9293503 | C | T | 0.1146 | -0.1653 | 0.0242 | 8.65E-12 |
| rs9318105 | A | G | 0.2427 | -0.1001 | 0.0176 | 1.34E-08 |
| rs9319731 | A | G | 0.1924 | -0.1214 | 0.0192 | 2.43E-10 |
| rs9364814 | T | C | 0.6125 | 0.0903 | 0.0155 | 5.96E-09 |
| rs9365555 | G | A | 0.3255 | -0.1006 | 0.0162 | 4.88E-10 |
| rs9367926 | G | C | 0.1735 | -0.125 | 0.02 | 4.40E-10 |
| rs9376090 | C | T | 0.2588 | -0.1149 | 0.0171 | 1.62E-11 |
| rs9419374 | G | A | 0.6468 | -0.1033 | 0.0166 | 4.62E-10 |
| rs9428015 | A | G | 0.6724 | -0.1078 | 0.0164 | 4.61E-11 |
| rs9456648 | T | C | 0.319 | -0.1003 | 0.0161 | 4.64E-10 |
| rs9467545 | T | A | 0.1569 | 0.2275 | 0.0206 | 1.81E-28 |
| rs9472135 | C | T | 0.3001 | -0.1443 | 0.0165 | 1.81E-18 |
| rs9477605 | A | G | 0.3526 | 0.0869 | 0.0157 | 3.22E-08 |
| rs9479196 | T | C | 0.1143 | -0.1841 | 0.0237 | 7.50E-15 |
| rs9508495 | T | C | 0.7561 | -0.187 | 0.0176 | 1.71E-26 |
| rs9532243 | C | A | 0.5176 | -0.1448 | 0.015 | 4.01E-22 |
| rs9532979 | G | A | 0.3063 | -0.0947 | 0.0163 | 6.66E-09 |
| rs954767 | C | A | 0.2595 | 0.1355 | 0.0171 | 2.06E-15 |
| rs9559749 | A | G | 0.218 | 0.1191 | 0.0185 | 1.11E-10 |
| rs9563529 | T | G | 0.2046 | 0.1198 | 0.0188 | 1.74E-10 |
| rs9572783 | G | C | 0.0814 | 0.1792 | 0.0277 | 9.35E-11 |
| rs9608637 | A | G | 0.5185 | -0.0977 | 0.0151 | 9.56E-11 |
| rs9609429 | C | T | 0.2734 | -0.108 | 0.0172 | 3.27E-10 |
| rs9646515 | G | A | 0.293 | 0.1207 | 0.0165 | 2.89E-13 |
| rs964941 | A | G | 0.5183 | 0.1481 | 0.0151 | 1.24E-22 |
| rs9649464 | A | G | 0.5592 | -0.0833 | 0.0151 | 3.74E-08 |
| rs9685837 | A | G | 0.3068 | -0.0923 | 0.0164 | 1.98E-08 |
| rs9841978 | A | G | 0.3255 | 0.167 | 0.0161 | 4.63E-25 |
| rs9844972 | C | G | 0.0715 | 0.1998 | 0.0305 | 6.11E-11 |
| rs9863881 | T | C | 0.4381 | 0.0831 | 0.015 | 3.28E-08 |
| rs9865843 | G | A | 0.4824 | 0.0913 | 0.0152 | 1.99E-09 |
| rs9875380 | T | C | 0.4626 | -0.0848 | 0.015 | 1.74E-08 |
| rs9878099 | G | C | 0.6288 | -0.093 | 0.0155 | 2.22E-09 |
| rs9890032 | G | C | 0.3847 | -0.1088 | 0.0155 | 2.30E-12 |
| rs9932220 | A | G | 0.2171 | -0.1336 | 0.0182 | 2.16E-13 |
| rs9937801 | C | T | 0.4302 | -0.1277 | 0.0151 | 2.15E-17 |
| rs9945516 | G | C | 0.3531 | 0.1024 | 0.0157 | 7.37E-11 |
| rs9967367 | T | C | 0.2981 | 0.1175 | 0.0166 | 1.45E-12 |
